# Effects of a Smartphone-Based Out-of-Hospital Screening App for Neonatal Hyperbilirubinemia on Neonatal Readmission Rates and Maternal Anxiety: Randomized Controlled Trial

**DOI:** 10.2196/37843

**Published:** 2022-11-23

**Authors:** Qin Yan, Yanhong Gong, Qing Luo, Xiaoxv Yin, Ling Yang, Honglin Wang, Juan Feng, Kaihui Xing, Yan Huang, Chuican Huang, Lichun Fan

**Affiliations:** 1 Department of Social Medicine and Health Management, Tongji Medical College, Huazhong University of Science and Technology Wuhan China; 2 Department of Child Heath Care, Hainan Women and Children’s Medical Center Haikou China; 3 Department of Child Health Care, National Center for Women and Children's Health, Chinese Center for Disease Control and Prevention Beijing China; 4 Department of Neonatology, Hainan Women and Children’s Medical Center Haikou China; 5 Obstetrical Department, Hainan Women and Children’s Medical Center Haikou China

**Keywords:** eHealth, mHealth, mobile apps, maternal anxiety, neonatal jaundice, neonatal readmission, neonatal screening, mobile phone

## Abstract

**Background:**

Neonatal hyperbilirubinemia is one of the leading causes of neonatal readmission—especially severe hyperbilirubinemia and its complications—and it influences disease burden as well as neonatal and maternal health. Smartphones have been shown to have satisfactory accuracy in screening neonatal bilirubin levels, but the impact of this technology on neonatal health care service and maternal health outcomes is still unknown.

**Objective:**

The aim of this study was to evaluate the impact of a smartphone-based out-of-hospital neonatal jaundice screening program on neonatal readmission rates for jaundice and related maternal anxiety.

**Methods:**

This was a 2-arm, unblinded, randomized controlled trial with 30 days of intervention and follow-up periods. From August 2019 to August 2020, healthy mother-infant dyads were recruited on-site from 3 public hospitals in Hainan, China. Intervention group mothers used the smartphone app to routinely monitor neonatal jaundice at home under the web-based guidance of pediatricians. Control group participants received routine care. The primary study outcome was the neonatal readmission rate due to jaundice within 30 days of the first hospital discharge. The secondary outcome was the maternal anxiety score associated with neonatal jaundice. The data were collected through a self-assessed questionnaire. All participants were included in the analysis (intention-to-treat).

**Results:**

In this study, 1424 mother-infant dyads were recruited, comprising 1424 mothers and 1424 newborns. The median age of the mothers was 29 (IQR 26-32) years, and there were 714 (50.1%) male neonates. These mother-infant dyads were randomly assigned to the intervention group and the control group, with 712 dyads in each group; only 1187 of these dyads completed the follow-up. We found that the adjusted 30-day neonatal readmission rate due to jaundice reduced by 10.5% (71/605, 11.7% vs 141/582, 24.2%; 95% CI 5%-15.9%; odds ratio 0.4, 95% CI 0.3-0.5; *P*<.001) and the relevant maternal anxiety mean score decreased by 3.6 (95% CI –4.4 to –2.8; β=–3.6, 95% CI –4.5 to –2.8; *P*<.001) in the intervention group compared to those in the routine care group.

**Conclusions:**

Our study shows that the smartphone-based out-of-hospital screening method for neonatal hyperbilirubinemia decreased the neonatal readmission rate within 30 days from the first discharge and improved maternal mental health to some degree, thus demonstrating the usefulness of this screening app for follow-up in pediatric care.

**Trial Registration:**

China Clinical Trial Registration Center, ChiCTR2100049567; http://www.chictr.org.cn/showproj.aspx?proj=64245

## Introduction

Neonatal hyperbilirubinemia (or neonatal jaundice) is a clinical condition in which the skin, sclera, and mucous membranes become yellowish as a result of elevated serum bilirubin levels caused by abnormalities in bilirubin metabolism [1]. It is a common condition in the neonatal period, particularly in the first 2 weeks of birth, and influences approximately 60%-80% of newborns globally [2]. About 8%-9% of infants are diagnosed with severe hyperbilirubinemia in the first week of life, which affects about 1.1 million newborns annually [3,4]. Some cases may develop into acute bilirubin encephalopathy or nuclear jaundice, causing irreversible neurological damage, and even threatening the life of the child [5,6]. Neonatal hyperbilirubinemia is one of the leading causes of neonatal readmission [7]. In most cases, neonatal jaundice is physiological and always subsides spontaneously [8], but patients with severe hyperbilirubinemia and its complications should be hospitalized [9]. In addition, neonatal jaundice not only increases neonatal pain but also raises the stress levels of the parents, especially the mother [7]. Coupled with their lack of knowledge about this disease and the nursing methods, this disease may have a great impact on infant rehospitalization [10,11].

Early identification of high-risk neonates, appropriate follow-ups, and timely intervention contribute to preventing the deterioration of neonatal hyperbilirubinemia [12], thereby reducing neonatal readmission to hospitals and the consumption of health care resources [13]. Although hyperbilirubinemia screening recommendations should be based on risk factors according to the American Academy of Pediatrics [14], they do not obviate the need for universal evaluation of jaundice in the neonatal period [15]. The increasingly short postpartum length of hospital stay of newborns make follow-up monitoring after discharge to home extremely important [16]. The gold standard for the diagnosis of neonatal jaundice is total serum bilirubin (TSB) levels, but it requires specialized testing equipment in the hospital, as invasive procedures increase infant pain [17]. Transcutaneous bilirubinometry (TcB) measurement has been proven to be feasible and noninvasive, but the high cost of TcB meters limit its wide application, particularly in out-of-hospital settings [18,19]. Therefore, it is usually difficult to perform TSB and TcB measurements for most families. Concurrently, the serious underfunding of the health workforce in most low-income countries [20] and the global shortage of health resources, especially pediatricians, have made postpartum follow-up carried out by health care professionals challenging. Therefore, it is necessary to investigate a convenient way to screen neonatal jaundice that does not entirely rely on medical professionals but can be performed by parents at home at any time.

Mobile communication devices have been widely used in the health care sector [21]. Such emerging information technologies may help overcome the barriers existing in the routine monitoring of neonatal jaundice. Several clinical trials have found that smartphone-based jaundice testing is accurate and cost-effective in identifying neonates with high levels of TSB [7,22-25]. However, mobile technology has not yet been formally used in jaundice screening practices. So far, there is no high-quality evidence to confirm the actual effectiveness of out-of-hospital jaundice screening by using smartphone apps, such as the effects on neonatal health care services and maternal health outcomes. On the basis of routine care, we have established a smartphone-based, family-physician collaborative mechanism for an out-of-hospital jaundice screening program. We performed a randomized controlled trial (RCT) to evaluate whether this smartphone-based intervention could reduce the 30-day neonatal readmission rates and maternal anxiety compared with the routine care.

## Methods

### Study Design

This study was an unblinded, parallel-assignment RCT conducted at 3 public hospitals in Hainan, China from August 2019 to September 2020. Participants were randomly assigned to either the smartphone-based intervention group or the routine care control group and were followed up for 30 days (Multimedia Appendix 1).

### Ethics Approval

The ethics committee of the Hainan Women and Children’s Medical Center in Hainan approved the trial protocol (institutional review board approval HNWCMC201605), and all participants provided written informed consent. The trial registration number is ChiCTR2100049567 in the China Clinical Trial Registration Center database.

### Study Participants

Neonates born in Hainan Women and Children’s Medical Center, Wanning People’s Hospital, and Chengmai People’s Hospital and their mothers were recruited as participants. All mother-infant dyads met the hospital discharge criteria as judged by the bedside doctor, and they were about to be discharged home. The eligible mother was at least 16 years old with clear understanding and communication skills, owned a smartphone, and was able to use it. Exclusion criteria included multiple births (eg, twins); neonates with congenital disease, perinatal asphyxia, neonatal infection, hemolytic disease (positive Coombs test), or other serious organ damage; or family not living in Hainan within the study period. Qualified mother-infant dyads were identified and screened face-to-face by trained obstetric nurses prior to discharge from the hospital. All enrolled persons were aware of the trial objective and process, and written informed consent was obtained from mothers at study entry.

### Randomization and Masking

After baseline information was collected, the trained obstetric nurses carried out simple randomization (draw of lots) and allocated each mother-infant dyad to the intervention and control groups. Because of the nature of the intervention, it was not possible to blind participants or the pediatric nurses who were responsible for participant recruitment and follow-up.

### Comparison Condition

Participants randomized to the control group received routine care. Under the National Basic Public Health Service Program in China [26], the primary health care institutions conduct newborn home visits (within 1 week of hospital discharge) and full-term health management for newborns (28-30 days after birth). Neonates with high risk factors such as low birth weight and prematurity are visited more frequently according to the actual situation.

### Intervention

Participants randomized to the intervention group received routine care and the smartphone-based, family-physician collaborative neonatal jaundice screening program, that is, under the remote guidance of a pediatrician, mothers routinely monitored neonatal jaundice at home by using a smartphone app. The jaundice mobile monitoring app used in this study is a publicly available, free downloadable software in China that allows remote, noninvasive, and self-service jaundice monitoring and early warnings. It is used in combination with a jaundice colorimetric card, which is required to be placed on the chest of the child. After judging the image quality, light environment, and skin area, the app will automatically scan and take photos to obtain a clear image of the newborn skin. Once the cloud server receives the image data, the jaundice value will be automatically calculated and displayed (automated image-based bilirubin test), along with an indication of the risk level of neonatal jaundice (low risk, 2.6-10.1 mg/L; medium risk, 10.2-17.1 mg/L; high risk: ≥17.2 mg/L). A clinical trial conducted to evaluate the screening accuracy of this app found strong concordance between automated image-based bilirubin test and TSB levels (*r*=0.97) [22].

Intervention group mothers received free appropriative jaundice colorimetric cards and installed the jaundice mobile monitoring app on their smartphones at hospital discharge. They were instructed by trained obstetric nurses about the data uploading method. They were required to detect neonatal bilirubin values by using the app, when needed, after returning home. We assigned each mother a pediatrician from the hospital where the delivery took place and bound the mother’s and the doctor’s versions of the jaundice mobile monitoring app together; both parties could use the app platform for communication and consultation. Pediatricians would be able to access the dynamic changes and risk prompts of neonatal bilirubin values synchronously, so as to judge the infants’ situation and guide the mother to take the correct preventive measures. If neonatal bilirubin level was considered to be at a high-risk level or the condition was quite severe, the pediatrician would alert the mother to bring the child to the hospital for further examination and specialist treatment. In addition, pediatricians would supervise and remind mothers to measure newborn infants’ jaundice level every day through the app.

### Outcome Measures

On the 31st day after the mother-infant dyad was discharged from the hospital, the maternal and neonatal outcomes were collected by trained obstetric nurses through telephone follow-up, and the last questionnaire survey was completed in September 2020. The primary outcome was the neonatal readmission rate due to jaundice, which was defined as the ratio of the number of neonates readmitted to hospitals for jaundice within 30 days of the first discharge to the total number of neonates in each group. Mothers were asked to answer, “Has your baby gone to the hospital again because of jaundice in the past 30 days?” and the corresponding options were yes or no. The secondary outcome was the maternal anxiety score associated with neonatal jaundice, which was measured by a self-designed scale (Multimedia Appendix 2). The scale consisted of 10 items with a 4-level scoring method and from “never happened” to “always happened;” the scores were coded with 1 to 4 points, respectively. The higher the total score (range 10-40 points), the more serious was the anxiety. It has good internal consistency, with Cronbach α=.91. Questions were answered by mothers whose neonates developed jaundice after discharge to home. In addition, the intervention group mothers were also asked to rate the jaundice mobile monitoring app in 5 aspects (ie, convenience, trustworthiness, recommendation, generalizability, and satisfaction) to assess the acceptability of the app. At recruitment, maternal and neonatal basic demographic characteristics were collected through a self-assessed questionnaire. Maternal anxiety score associated with neonatal jaundice was also included, and the assessment tool was the same as that used for the secondary outcome. Mothers whose neonates developed jaundice at baseline could respond to that scale.

### Sample Size

A sample size calculation was performed based on the primary outcome. Based on previous studies [27,28] and our hypothesis that the 30-day neonatal readmission rate due to jaundice would relatively reduce by 50% in this trial as well as considering about 10% attrition rate, we estimated that about 600 neonates were needed for each group to ensure that the difference could be detected at 80% power (α=.05, 2-sided), that is, there was a total requirement of 1200 mother-infant dyads at least.

### Statistical Analysis

All analyses followed the intention-to-treat principle, including all the randomly assigned mother-infant dyads. We examined the distributions of the baseline characteristics by using descriptive statistics and Pearson chi-square test; 2-sided Student *t* test and Wilcoxon rank sum test were applied to compare the difference between the 2 groups. We conducted analyses using a binary logistic regression model for primary outcomes and a multiple linear regression model for secondary outcomes and reported the odds ratio (OR), regression coefficient (β), and 95% CIs. We adjusted all the models for observed baseline maternal and neonatal characteristics (ie, maternal age, nationality, education level, employment status, medical insurance, residence, convenience of access to health care, economic status and neonatal gender, gestational age, birth weight, feeding patterns, parity, status of jaundice before discharge, and delivery method). We added some post hoc analyses. First, based on least squares regression and identity link function, the generalized linear models were used to enable estimation of the adjusted risk difference for binary outcomes and adjusted mean difference for continuous outcomes. Besides, we fitted a linear mixed-effects repeated measures model to maternal anxiety scores at baseline and follow-up, thus capturing whether the improvement in the smartphone-based intervention group was greater than that in the control group. Sensitivity analyses were performed to assess the robustness of the primary and secondary outcome analysis. We performed multivariate imputation by using chained equations to impute all missing data, including outcomes that were not followed up. All statistical analyses were conducted using SAS version 9.4 (SAS Institute) and R version 4.0.3 (R Development Core Team), and all hypothesis tests were 2-sided with a significance level of .05*.*

## Results

### Overview

**Figure 1:**
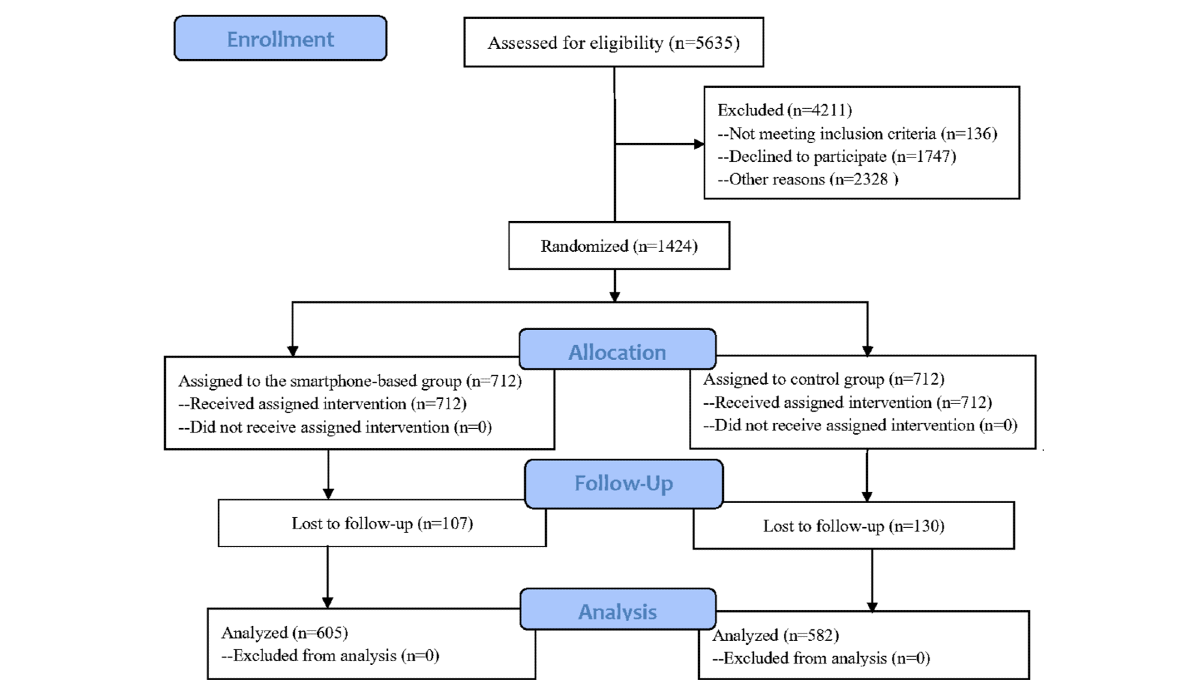
Consolidated Standards of Reporting Trials (CONSORT) diagram for this study.

From August 2019 to August 2020, we screened 5635 mother-infant dyads in 3 public hospitals and 1424 dyads were eligible (Figure 1). Among them, 712 were randomly classified into the smartphone-based intervention group and 712 into the routine-care control group. On the 31st day of the intervention, 605 intervention group and 582 control group mother-infant dyads were followed up, as they were available for the analysis; others were lost because of refusal to follow-up, dropouts, etc, with a total retention rate of 83.4% (1187/1424).

### Baseline Data

The median age of the mothers was 29 (IQR 26-32) years; 32.3% (460/1424) of the mothers lived in the rural areas, and the mean maternal anxiety score associated with neonatal jaundice was 19.6 (SD 6.3) (Table 1). Of the 1424 neonates, 714 (50.1%) were males, 899 (63.1%) were born through natural delivery, and 1062 (74.6%) were breastfed (Table 2). The baseline characteristics were well-balanced between the control and the intervention groups. Moreover, similar demographic characteristics were observed between those who completed this trial and those who did not complete this trial (Multimedia Appendix 3). 

**Table 1:** Baseline characteristics of the mothers.^a^

Variables			Overall (N=1424)		Intervention group (n=712）		Control group (n=712)
Age (years), median (IQR)			29 (26-32)		29 (26-32)		29 (26-32)
**Nationality^b^, n (%)**							
	Han nationality	1338 (94.1)		670 (94.4)		668 (93.8)	
	Minority	84 (5.9)		40 (5.6)		44 (6.2)	
**Education level, n (%)**							
	Junior high school or below	376 (26.4)		190 (26.7)		186 (26.1)	
	High school or technical secondary school	332 (23.3)		167 (23.5)		165 (23.2)	
	Junior college	338 (23.7)		170 (23.9)		168 (23.6)	
	Undergraduate or above	378 (26.5)		185 (26)		193 (27.1)	
**Employment status^c^, n (%)**							
	Formally employed	634 (44.6)		315 (44.3)		319 (44.8)	
	Temporary employed	77 (5.4)		33 (4.6)		44 (6.2)	
	Unemployed	712 (50)		363 (51.1)		349 (49.1)	
**Medical insurance^d^, n (%)**							
	Yes	1254 (88.2)		621 (87.5)		633 (88.9)	
	No	168 (11.8)		89 (12.5)		79 (11.1)	
**Residence, n (%)**							
	Urban	964 (67.7)		479 (67.3)		485 (68.1)	
	Rural	460 (32.3)		233 (32.7)		227 (31.9)	
**Convenience of access to health care^e^, n (%)**							
	Yes	1246 (87.6)		616 (86.8)		630 (88.5)	
	No	176 (12.4)		94 (13.2)		82 (11.5)	
**Economic status^f^, n (%)**							
	High income	51 (3.6)		25 (3.5)		68 (9.6)	
	Middle income	1230 (86.5)		612 (86.1)		618 (86.8)	
	Low income	142 (10)		74 (10.4)		26 (3.7)	
Maternal anxiety score^g^, mean (SD)			19.61 (6.3)		19.79 (6.3)		19.42 (6.3)

^a^*P* values were obtained using 2-sided Student *t* test or Wilcoxon rank sum test for continuous and *χ*^2^ test for categorical variables; all *P*>.05.

^b^Nationality was unknown for 2 intervention group mothers.

^c^Employment status was unknown for 1 intervention group mother.

^d^Medical insurance was unknown for 2 intervention group mothers.

^e^Convenience of access to health care represents self-assessed convenience of access to health care facilities from residence by mothers; this was unknown for 2 intervention group mothers.

^f^Economic status was unknown for 1 intervention group mother.

^g^Including mothers whose children developed jaundice at baseline (604 in the intervention group, 608 in the control group, as shown in Table 2).

**Table 2:** Baseline characteristics of the neonates.^a^

Variables			Overall (N=1424)		Intervention group (n=712）		Control group (n=712)
**Gender, n (%)**							
	Male	714 (50.1)		362 (50.8)		352 (49.4)	
	Female	710 (49.9)		350 (49.2)		360 (50.6)	
Gestational age^b^ (weeks), median (IQR)			39.1 (38.57-39.9)		39.1 (38.43-39.86)		39.1 (38.6-40.0)
Birth weight^c^ (grams), median (IQR)			3200 (2900-3450)		3150 (2950-3450)		3200 (2900-3450)
**Feeding patterns^d^, n (%)**							
	Breastfeeding	1062 (74.6)		523 (73.5)		539 (75.8)	
	Nonbreastfeeding	28 (2)		13 (1.8)		15 (2.1)	
	Mixed feeding	333 (23.4)		176 (24.7)		157 (22.1)	
**Parity, n (%)**							
	First child	630 (44.2)		317 (44.5)		313 (44)	
	Second child	677 (47.5)		338 (47.5)		339 (47.6)	
	Third child or later	117 (8.2)		57 (8)		60 (8.4)	
**Status of jaundice before discharge^e^, n (%)**							
	Not present	211 (14.8)		108 (15.2)		103 (14.5)	
	Present	1212 (85.2)		604 (84.8)		608 (85.5)	
**Delivery method, n (%)**							
	Natural delivery	899 (63.1)		476 (66.9)		482 (67.7)	
	Cesarean section	466 (32.7)		236 (33.2)		230 (32.3)	

^a^*P* values were obtained using 2-sided Student *t* test or Wilcoxon rank sum test for continuous and *χ*^2^ test for categorical variables; all *P*>.05.

^b^Gestational age was unknown for 1 intervention group neonate and 1 control group neonate.

^c^Birth weight was unknown for 2 intervention group neonates and 2 control group neonates.

^d^Feeding pattern was unknown for 1 control group neonate.

^e^Status of jaundice before discharge represents if the neonate developed jaundice at baseline; this was unknown for 1 control group neonate.

### Primary Outcome

Within 30 days of the first hospital discharge, 68.1% (412/605) and 70.1% (408/582) of the neonates in the intervention and control groups, respectively, who were successfully followed up showed jaundice symptoms, with no statistical difference between the 2 groups. In comparison with the control group, the smartphone-based intervention group was significantly associated with a decrease (141/582, 24.2% vs 71/605, 11.7%, respectively; risk difference=12.5%, 95% CI 8.2%-16.8%) in the neonatal readmission rate due to jaundice (OR 0.4, 95% CI 0.3-0.6). After adjusting the model, our intervention remained observably effective in reducing the risk of readmission (OR 0.4, 95% CI 0.3-0.5), although the degree of reduction was smaller than before (risk difference=10.5%, 95% CI 5%-15.9%) (Table 3).

**Table 3:** Differences in the primary and secondary outcomes between the 2 groups.

Outcomes	Intervention group (n=605)	Control group (n=582)	Unadjusted		Adjusted	
			Odds ratio (95% CI) or β (95% CI)	Difference^a^ (95% CI)	Odds ratio (95% CI) or β (95% CI)	Difference^a^ (95% CI)
Primary outcome, n (%)	71 (11.7)	141 (24.2)	0.4 (0.3 to 0.6)^b^	12.5% (8.2% to 16.8%)	0.4 (0.3 to 0.5)^b^	10.5% (5% to 15.9%)
Secondary outcome^c^, mean (SD)	16.8 (4.2)	20.5 (7.4)	–3.7 (–4.6 to –2.9)^b^	–3.7 (–4.6 to –2.9)	–3.6 (–4.5 to –2.8)^b^	–3.6 (–4.4 to –2.8)

^a^Difference represents risk difference for primary outcome and mean difference for secondary outcome. Risk difference represents the absolute value of the difference in the neonatal readmission rates between the 2 groups. Mean difference represents the difference in the mean maternal anxiety scores between the 2 groups.

^b^*P*<.001 *P* values were from the odds ratio of the binary logistic regression model and β values were of the multiple linear regression model, with the control group as reference. The primary outcome was neonatal readmission. The secondary outcome was maternal anxiety score due to neonatal jaundice.

^c^Including mothers whose children developed jaundice symptoms after hospital discharge (intervention group, n=412; control group, n=408).

### Secondary Outcome

**Figure 2:**
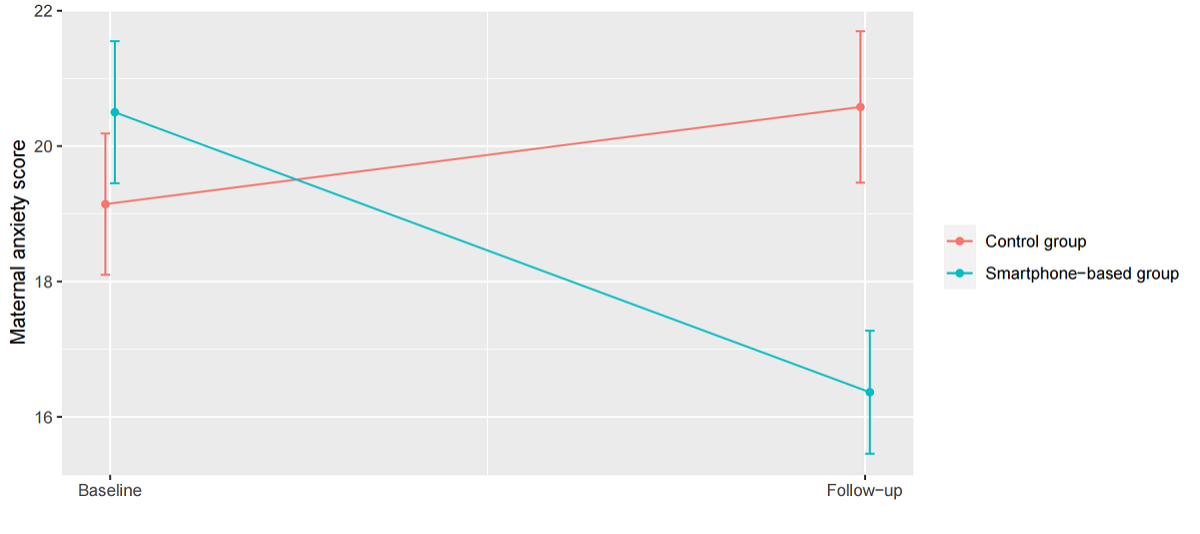
Changes in the adjusted mean maternal anxiety score from baseline to the 31st day of follow-up (n=695). Points and error bars show the least squares mean and 95% CIs, respectively, that were derived from the adjusted linear mixed-effects repeated measures model.

Maternal anxiety scores associated with neonatal jaundice in the smartphone-based intervention group were apparently lower than those in the control group (mean difference=–3.7, 95% CI –4.6 to –2.9). The adjusted model showed a similar intervention effect (mean difference=–3.6, 95% CI –4.5 to –2.8) (Table 3). We performed a post hoc analysis to examine whether improvement in maternal anxiety could be explained by underlying temporal trends. There were 347 neonates in the intervention group and 348 in the control group who developed jaundice both at baseline and during the intervention period follow-up. For these participants, maternal anxiety is shown in Figure 2. From baseline to the 31st day of follow-up, the mean maternal anxiety score in the intervention group reduced by 4.1 (95% CI –4.9 to 3.4); however, that in the control group slightly increased by 1.4 (95% CI 0.5-2.4). After follow-up, the maternal anxiety score in the intervention group was significantly lower than that in the control group (adjusted difference=–4.2, 95% CI –5.1 to –3.3). The results in the sensitivity analysis were similar to the above main results (Multimedia Appendix 4).

### Evaluation of the Jaundice Mobile Monitoring App

More than half of intervention group mothers rated the jaundice mobile monitoring app as relatively or very convenient (331/580, 57.1%) and relatively or very credible (295/597, 51%) in measuring their child’s jaundice. Approximately 87.1% (504/579) of the mothers were willing to recommend this app to people in need, and 87.7% (508/579) held a positive attitude toward the necessity of promoting this app on a wide scale. In terms of satisfaction, only 11.1% (64/579) of the mothers showed dissatisfaction with the app (Multimedia Appendix 5).

## Discussion

### Principal Findings

In this smartphone-based RCT, we found that the intervention had a significant impact on reducing the neonatal readmission for jaundice within 30 days of the initial discharge, wherein the readmission rate in the intervention group was 10.5% (95% CI 5%-15.9%) lower than that in the control group. In contrast with those in the control group, maternal anxiety symptoms induced by neonatal jaundice were also less severe in mothers who had installed and used the jaundice mobile monitoring app. Following-up monitoring of neonatal bilirubin levels is conducive to improving the outcome of neonatal hyperbilirubinemia, including reducing readmission rates [13,29,30]. One RCT implemented in China suggested that the neonatal readmission rate for hyperbilirubinemia was lower when neonatal TcB was regularly monitored by neonatologists compared with the control group wherein regular monitoring was absent (1.3% vs 3.3%, respectively) [31]. Another Australian study used TcB screening and visual assessment to detect the bilirubin levels of apparently healthy newborns before discharge and found that the former was capable of controlling neonatal readmission risk for hyperbilirubinemia (risk ratio 0.25, 95% CI 0.14-0.46) [27]. These studies were mostly based on TcB measurements, wherein parents and infants were asked to head to the follow-up clinic of the hospital or primary health care center on a specified date. The smartphone-based intervention in this trial emphasized more on the screening for neonatal jaundice at home, which may decrease the physical fatigue in mothers caused by repeated visits to the hospital. Our finding was consistent with the above studies basically [27,31], with positive effects in lessening neonatal readmission.

According to some surveys, the shorter the average length of postpartum hospital stay, the higher was the risk of 30-day neonatal readmission [32-34]. One possible reason for this is that both the time spent on neonatal health monitoring and the time parents received guidance from health care professionals are shortened due to the reduced length of postpartum hospital stay, with the consequence that parents may not have sufficient knowledge and confidence to cope with child's various health problems and thus increasing the corresponding health investment, such as hospital readmission [33]. Neonatal bilirubin levels usually peak at 96 hours of life when most infants have returned home, and jaundice symptoms may appear again after subsiding [35]. Nearly 70% of the newborns in our study developed jaundice after hospital discharge. Therefore, subsequent screening of neonatal jaundice is extremely necessary for mother and child. The smartphone-based intervention allowed mothers to observe the child’s bilirubin levels at home at any time and acquire professional web-based guidance from pediatricians, which played an essential role in boosting parental confidence and thus avoiding some unnecessary hospital admissions.

Our intervention has proved to be effective in maintaining maternal mental health. Other studies have shown that family-centered parent-involved daily neonatal health care is positively associated with clinical benefits for infants (decreased readmission rate) and improved parental outcomes (decreased anxiety, depression, and stress) [36,37]. In addition, reducing neonatal readmission rate prevents separation of the mother and infant, thereby relieving the maternal emotional stress associated with it [13]. The mother’s psychological status, in turn, affects the risk of infant rehospitalization. A previous study has shown that mothers with high anxiety traits were nearly 3 times more likely to take their babies to the hospital than their counterparts [11]. Thus, psychosocial support for mothers could reduce the readmission rates of healthy newborns [38]. The intervention in this study eased maternal negative emotions in the face of neonatal jaundice, which might control newborn readmission indirectly and allow them to experience less pain. It should be noted that mothers who did not receive the intervention had significantly higher anxiety scores after hospital discharge compared to baseline, which possibly, in part, could be because they were away from their health care providers and not able to keep track of the child’s health accurately. This indicates that we should enhance the maternal awareness of neonatal jaundice and provide them with adequate psychological comfort in the future management of neonatal hyperbilirubinemia.

The application of the smartphone app in the monitoring of neonatal jaundice fits in with the widespread use of mobile technology currently; the global internet usage rate has reached 65.6% as of March 31, 2021 [39]. The previous cost-benefit analysis found that for every ¥1 invested in the smartphone-based out-of-hospital screening program for neonatal jaundice, ¥18.76 was saved in the treatment cost of postdischarge neonatal jaundice (the average conversion rate at the time of the study in 2021 was US $1=¥6.4515) [40]. The low cost and high accessibility of the jaundice mobile monitoring app make this technology feasible for patients with medical conditions and lack of access to treatment in resource-poor regions. Moreover, this family-centered jaundice monitoring app may contribute to alleviating the shortage of pediatric specialists, thereby promoting the implementation of a basic public health service program.

Although this jaundice mobile monitoring app can be regarded as a good auxiliary screening tool for neonatal jaundice in some cases, there is greater scope for improving its acceptability, because the proportion of mothers who explicitly expressed satisfaction with the app was less than 50%. The potential reason for this low satisfaction rate may be that the app was developed by a non–health care provider, leading to some concerns about the accuracy and credibility of its information. In this regard, we suggest that on the premise of ensuring the quality of jaundice screening technology, telemedicine guidance from health care professionals should be facilitated and related units should optimize their publicity and promotion strategies.

### Strengths and Limitations

This study, to our knowledge, is the first RCT to analyze the effects of a smartphone app on neonatal health resource utilization and maternal mental health. Another strength of this study is the inclusion of large samples and enough statistical power to test the effects of the intervention.

Although our research results have important clinical implications, several limitations should be recognized. First, as we did not restrict the hospitals to which participants were readmitted, it was not possible to obtain and check all neonates’ visit records in the 3 study hospitals; therefore, self-reported data were uniformly used in all outcomes. Although some recall bias was inevitable, the short follow-up period (only 30 days) and the fact that parents usually attach importance to neonatal health and remember relatively well whether their child visited a doctor again minimized the recall bias. Second, due to the unavailability of the return visit records, we could not assess whether our intervention increased the readmission rates related to worsening neonatal hyperbilirubinemia, despite a significant drop in the overall rate. However, our intervention approaches are unlikely to delay admission to hospital for serious jaundice under the guidance of pediatricians. Finally, the trial was limited by the study site in comparison to that in other reports [27,28,31]. The 30-day neonatal readmission rate due to jaundice was higher in our study compared to that reported in previous studies [13,27,28,41]. This is partly due to inconsistent postdischarge follow-up periods across studies and due to the high prevalence of neonatal hyperbilirubinemia in Hainan, China—a region with a population with severe erythrocyte glucose-6-phosphate dehydrogenase deficiency [42,43]. This factor may affect the applicability of our findings to other populations. Therefore, it is necessary to validate the effectiveness of this intervention in a larger sample or in a cross-cultural context.

### Conclusion

Our study showed that a smartphone-based jaundice screening app was effective for decreasing the risk of neonatal readmission due to jaundice within 30 days of discharge from hospital and the related maternal anxiety. Smartphones are becoming increasingly used around the world widely, as they are highly accessible and can act as low-cost, self-service testing tools for measuring neonatal bilirubin levels. More studies are needed to analyze the effectiveness of this intervention and to ensure large-scale implementation in other settings, subject to adequate resources.

## Appendix

Multimedia Appendix 1 CONSORT-eHEALTH checklist (V 1.6.1).

Multimedia Appendix 2 Items of the self-designed maternal anxiety due to neonatal jaundice scale.

Multimedia Appendix 3 Differences in the demographic characteristics between participants who completed and did not complete the trial.

Multimedia Appendix 4 Sensitivity analyses.

Multimedia Appendix 5 Evaluation of the jaundice mobile monitoring app.

## Abbreviations

OR: odds ratio
RCT: randomized controlled trial
TcB: transcutaneous bilirubinometry
TSB: total serum bilirubin
