# Supplementary material for: Effects of a Smartphone-Based Out-of-Hospital Screening App for Neonatal Hyperbilirubinemia on Neonatal Readmission Rates and Maternal Anxiety: Randomized Controlled Trial
Source: J Med Internet Res. 2022 Nov 23;24(11):e37843. doi: 10.2196/37843 (PMC9730202; doi:10.2196/37843)
Supplement: Multimedia Appendix 1 [file jmir_v24i11e37843_app1.pdf]

CONSORT-EHEALTH (V 1.6.1) - Submission/Publication Form

The CONSORT-EHEALTH checklist is intended for authors of randomized trials evaluating web-based and Internet-based applications/interventions, including mobile interventions, electronic games (incl multiplayer games), social media, certain telehealth applications, and other interactive and/or networked electronic applications. Some of the items (e.g. all subitems under item 5 - description of the intervention) may also be applicable for other study designs.

The goal of the CONSORT EHEALTH checklist and guideline is to be
a) a guide for reporting for authors of RCTs,
b) to form a basis for appraisal of an ehealth trial (in terms of validity)

CONSORT-EHEALTH items/subitems are MANDATORY reporting items for studies published in the Journal of Medical Internet Research and other journals / scientific societies endorsing the checklist.

Items numbered 1., 2., 3., 4a., 4b etc are original CONSORT or CONSORT-NPT (non-pharmacologic treatment) items.
Items with Roman numerals (i., ii, iii, iv etc.) are CONSORT-EHEALTH extensions/clarifications.

As the CONSORT-EHEALTH checklist is still considered in a formative stage, we would ask that you also RATE ON A SCALE OF 1-5 how important/useful you feel each item is FOR THE PURPOSE OF THE CHECKLIST and reporting guideline (optional).

Mandatory reporting items are marked with a red *.
In the textboxes, either copy & paste the relevant sections from your manuscript into this form - please include any quotes from your manuscript in QUOTATION MARKS,
or answer directly by providing additional information not in the manuscript, or elaborating on why the item was not relevant for this study.

YOUR ANSWERS WILL BE PUBLISHED AS A SUPPLEMENTARY FILE TO YOUR PUBLICATION IN JMIR AND ARE CONSIDERED PART OF YOUR PUBLICATION (IF ACCEPTED).
Please fill in these questions diligently. Information will not be copyedited, so please use proper spelling and grammar, use correct capitalization, and avoid abbreviations.

DO NOT FORGET TO SAVE AS PDF _AND_ CLICK THE SUBMIT BUTTON SO YOUR ANSWERS ARE IN OUR DATABASE !!!

Citation Suggestion (if you append the pdf as Appendix we suggest to cite this paper in the caption):
Eysenbach G, CONSORT-EHEALTH Group
CONSORT-EHEALTH: Improving and Standardizing Evaluation Reports of Web-based and Mobile Health Interventions
J Med Internet Res 2011;13(4):e126

URL: http://www.jmir.org/2011/4/e126/
doi: 10.2196/jmir.1923
PMID: 22209829

登录 Google 即可保存进度。了解详情

*必填

Your name *

First Last

Yanhong Gong

Primary Affiliation (short), City, Country *

University of Toronto, Toronto, Canada

Department of Social Medicine and Health Ma

Your e-mail address *

abc@gmail.com

gongyanhong@hust.edu.cn

Title of your manuscript *

Provide the (draft) title of your manuscript.

Out-of-Hospital Screening for Neonatal Hyperbilirubinemia Using a Smartphone App: a Randomized Controlled Trial

Name of your App/Software/Intervention *

If there is a short and a long/alternate name, write the short name first and add the long name in brackets.

Jaundice Mobile Monitoring App/the smartphc

Evaluated Version (if any)

e.g. "V1", "Release 2017-03-01", "Version 2.0.27913"

您的回答

Language(s) *

What language is the intervention/app in? If multiple languages are available, separate by comma (e.g. "English, French")

Chinese

URL of your Intervention Website or App

e.g. a direct link to the mobile app on app in appstore (itunes, Google Play), or URL of the website. If the intervention is a DVD or hardware, you can also link to an Amazon page.

您的回答

URL of an image/screenshot (optional)

您的回答

Accessibility *

Can an enduser access the intervention presently?

- ○ access is free and open
- ○ access only for special usergroups, not open
- ◉ access is open to everyone, but requires payment/subscription/in-app purchases
- ○ app/intervention no longer accessible
- ○ 其他:

Primary Medical Indication/Disease/Condition *

e.g. "Stress", "Diabetes", or define the target group in brackets after the condition, e.g. "Autism (Parents of children with)", "Alzheimers (Informal Caregivers of)"

Neonatal Hyperbilirubinemia

Primary Outcomes measured in trial *

comma-separated list of primary outcomes reported in the trial

the neonatal readmission rate due to jaundice

Secondary/other outcomes

Are there any other outcomes the intervention is expected to affect?

the maternal anxiety score associated with neonatal jaundice

Recommended "Dose" *

What do the instructions for users say on how often the app should be used?

- ○ Approximately Daily
- ○ Approximately Weekly
- ○ Approximately Monthly
- ○ Approximately Yearly
- ◉ "as needed"
- ○ 其他:

Approx. Percentage of Users (starters) still using the app as recommended after 3 months *

- ◉ unknown / not evaluated
- ○ 0-10%
- ○ 11-20%
- ○ 21-30%
- ○ 31-40%
- ○ 41-50%
- ○ 51-60%
- ○ 61-70%
- ○ 71%-80%
- ○ 81-90%
- ○ 91-100%
- ○ 其他:

Overall, was the app/intervention effective? *

- (selected) yes: all primary outcomes were significantly better in intervention group vs control
- partly: SOME primary outcomes were significantly better in intervention group vs control
- no statistically significant difference between control and intervention
- potentially harmful: control was significantly better than intervention in one or more outcomes
- inconclusive: more research is needed
- 其他:

Article Preparation Status/Stage *

At which stage in your article preparation are you currently (at the time you fill in this form)

- not submitted yet - in early draft status
- not submitted yet - in late draft status, just before submission
- submitted to a journal but not reviewed yet
- (selected) submitted to a journal and after receiving initial reviewer comments
- submitted to a journal and accepted, but not published yet
- published
- 其他:

Journal *

If you already know where you will submit this paper (or if it is already submitted), please provide the journal name (if it is not JMIR, provide the journal name under "other")

- ○ not submitted yet / unclear where I will submit this
- ◉ Journal of Medical Internet Research (JMIR)
- ○ JMIR mHealth and UHealth
- ○ JMIR Serious Games
- ○ JMIR Mental Health
- ○ JMIR Public Health
- ○ JMIR Formative Research
- ○ Other JMIR sister journal
- ○ 其他:

Is this a full powered effectiveness trial or a pilot/feasibility trial? *

- ○ Pilot/feasibility
- ◉ Fully powered

Manuscript tracking number *

If this is a JMIR submission, please provide the manuscript tracking number under "other" (The ms tracking number can be found in the submission acknowledgement email, or when you login as author in JMIR. If the paper is already published in JMIR, then the ms tracking number is the four-digit number at the end of the DOI, to be found at the bottom of each published article in JMIR)

- ○ no ms number (yet) / not (yet) submitted to / published in JMIR
- ◉ 其他: 37843

TITLE AND ABSTRACT

1a) TITLE: Identification as a randomized trial in the title

1a) Does your paper address CONSORT item 1a? *

I.e does the title contain the phrase "Randomized Controlled Trial"? (if not, explain the reason under "other")

- (●) yes
- ( ) 其他:

1a-i) Identify the mode of delivery in the title

Identify the mode of delivery. Preferably use “web-based” and/or “mobile” and/or “electronic game” in the title. Avoid ambiguous terms like “online”, “virtual”, “interactive”. Use “Internet-based” only if Intervention includes non-web-based Internet components (e.g. email), use “computer-based” or “electronic” only if offline products are used. Use “virtual” only in the context of “virtual reality” (3-D worlds). Use “online” only in the context of “online support groups”. Complement or substitute product names with broader terms for the class of products (such as “mobile” or “smart phone” instead of “iphone”), especially if the application runs on different platforms.

	1	2	3	4	5	
subitem not at all important	( )	( )	( )	( )	(●)	essential

清除所选内容

Does your paper address subitem 1a-i? *

Copy and paste relevant sections from manuscript title (include quotes in quotation marks "like this" to indicate direct quotes from your manuscript), or elaborate on this item by providing additional information not in the ms, or briefly explain why the item is not applicable/relevant for your study

Yes, like this “Out-of-Hospital Screening for Neonatal Hyperbilirubinemia Using a Smartphone App: a Randomized Controlled Trial”

1a-ii) Non-web-based components or important co-interventions in title

Mention non-web-based components or important co-interventions in title, if any (e.g., “with telephone support”).

	1	2	3	4	5	
subitem not at all important	○	○	○	○	○	essential

Does your paper address subitem 1a-ii?

Copy and paste relevant sections from manuscript title (include quotes in quotation marks "like this" to indicate direct quotes from your manuscript), or elaborate on this item by providing additional information not in the ms, or briefly explain why the item is not applicable/relevant for your study

Not applicable. There were not involved non-web-based components or important co-interventions in our reaearch.

1a-iii) Primary condition or target group in the title

Mention primary condition or target group in the title, if any (e.g., “for children with Type I Diabetes”) Example: A Web-based and Mobile Intervention with Telephone Support for Children with Type I Diabetes: Randomized Controlled Trial

	1	2	3	4	5	
subitem not at all important	○	○	○	○	○	essential

Does your paper address subitem 1a-iii? *

Copy and paste relevant sections from manuscript title (include quotes in quotation marks "like this" to indicate direct quotes from your manuscript), or elaborate on this item by providing additional information not in the ms, or briefly explain why the item is not applicable/relevant for your study

Yes, like this “Out-of-Hospital Screening for Neonatal Hyperbilirubinemia Using a Smartphone App: a Randomized Controlled Trial”

1b) ABSTRACT: Structured summary of trial design, methods, results, and conclusions

NPT extension: Description of experimental treatment, comparator, care providers, centers, and blinding status.

1b-i) Key features/functionalities/components of the intervention and comparator in the METHODS section of the ABSTRACT

Mention key features/functionalities/components of the intervention and comparator in the abstract. If possible, also mention theories and principles used for designing the site. Keep in mind the needs of systematic reviewers and indexers by including important synonyms. (Note: Only report in the abstract what the main paper is reporting. If this information is missing from the main body of text, consider adding it)

	1	2	3	4	5	
subitem not at all important	○	○	○	○	○	essential

Does your paper address subitem 1b-i? *

Copy and paste relevant sections from the manuscript abstract (include quotes in quotation marks "like this" to indicate direct quotes from your manuscript), or elaborate on this item by providing additional information not in the ms, or briefly explain why the item is not applicable/relevant for your study

Yes, like this “This was a two-arm, unblinded, randomized controlled trial with 30 days of intervention and follow-up periods. From August 2019 to August 2020, healthy mother-infant dyads were recruited on site from three public hospitals in Hainan Province, China. Intervention group mothers used the smartphone APP to routinely monitor neonatal jaundice at home under the online guidance of paediatricians. Control group participants receive routine care.”

1b-ii) Level of human involvement in the METHODS section of the ABSTRACT

Clarify the level of human involvement in the abstract, e.g., use phrases like “fully automated” vs. “therapist/nurse/care provider/physician-assisted” (mention number and expertise of providers involved, if any). (Note: Only report in the abstract what the main paper is reporting. If this information is missing from the main body of text, consider adding it)

	1	2	3	4	5	
subitem not at all important	○	○	○	○	○	essential

Does your paper address subitem 1b-ii?

Copy and paste relevant sections from the manuscript abstract (include quotes in quotation marks "like this" to indicate direct quotes from your manuscript), or elaborate on this item by providing additional information not in the ms, or briefly explain why the item is not applicable/relevant for your study

Yes, like this “Intervention group mothers used the smartphone APP to routinely monitor neonatal jaundice at home under the online guidance of paediatricians.”

1b-iii) Open vs. closed, web-based (self-assessment) vs. face-to-face assessments in the METHODS section of the ABSTRACT

Mention how participants were recruited (online vs. offline), e.g., from an open access website or from a clinic or a closed online user group (closed usergroup trial), and clarify if this was a purely web-based trial, or there were face-to-face components (as part of the intervention or for assessment). Clearly say if outcomes were self-assessed through questionnaires (as common in web-based trials). Note: In traditional offline trials, an open trial (open-label trial) is a type of clinical trial in which both the researchers and participants know which treatment is being administered. To avoid confusion, use “blinded” or “unblinded” to indicated the level of blinding instead of “open”, as “open” in web-based trials usually refers to “open access” (i.e. participants can self-enrol). (Note: Only report in the abstract what the main paper is reporting. If this information is missing from the main body of text, consider adding it)

	1	2	3	4	5	
subitem not at all important	○	○	○	○	○	essential

Does your paper address subitem 1b-iii?

Copy and paste relevant sections from the manuscript abstract (include quotes in quotation marks "like this" to indicate direct quotes from your manuscript), or elaborate on this item by providing additional information not in the ms, or briefly explain why the item is not applicable/relevant for your study

Yes, like this “From August 2019 to August 2020, healthy mother-infant dyads were recruited on site from three public hospitals in Hainan Province, China.” “The data was collected through self-assessed questionnaire.”

1b-iv) RESULTS section in abstract must contain use data

Report number of participants enrolled/assessed in each group, the use/uptake of the intervention (e.g., attrition/adherence metrics, use over time, number of logins etc.), in addition to primary/secondary outcomes. (Note: Only report in the abstract what the main paper is reporting. If this information is missing from the main body of text, consider adding it)

	1	2	3	4	5	
subitem not at all important	○	○	○	○	○	essential

Does your paper address subitem 1b-iv?

Copy and paste relevant sections from the manuscript abstract (include quotes in quotation marks "like this" to indicate direct quotes from your manuscript), or elaborate on this item by providing additional information not in the ms, or briefly explain why the item is not applicable/relevant for your study

Yes, like this “1424 mother-infant dyads were recruited, comprising 1424 mothers and 1424 newborns [maternal median age was 29 (IQR, 29-32) years, 714 (50.1%) male neonates]. 1424 mother-infant dyads were randomly assigned to the intervention group and the control group, with 712 dyads in each group. A total of 1187 them completing follow-up. Compared with routine-care group, the adjusted 30-day neonatal readmission rate due to jaundice reduced 10.5% (11.7% vs 24.2%, 95%CI, -15.9% to -5.0%; OR, 0.4, 95%CI, 0.3 to 0.6, P < .001) and the relevant maternal anxiety mean score decreased 3.6 (95%CI, -4.4 to -2.8; β, -3.6, 95% CI, -4.5 to -2.8; P < .001).”

1b-v) CONCLUSIONS/DISCUSSION in abstract for negative trials

Conclusions/Discussions in abstract for negative trials: Discuss the primary outcome - if the trial is negative (primary outcome not changed), and the intervention was not used, discuss whether negative results are attributable to lack of uptake and discuss reasons. (Note: Only report in the abstract what the main paper is reporting. If this information is missing from the main body of text, consider adding it)

	1	2	3	4	5	
subitem not at all important	○	○	○	○	○	essential

Does your paper address subitem 1b-v?

Copy and paste relevant sections from the manuscript abstract (include quotes in quotation marks "like this" to indicate direct quotes from your manuscript), or elaborate on this item by providing additional information not in the ms, or briefly explain why the item is not applicable/relevant for your study

Yes, like this “The smartphone-based out-of-hospital screening method for neonatal hyperbilirubinemia decreased neonatal readmission rate within 30 days of first discharge and improved maternal mental health in some degree.”

INTRODUCTION

2a) In INTRODUCTION: Scientific background and explanation of rationale

2a-i) Problem and the type of system/solution

Describe the problem and the type of system/solution that is object of the study: intended as stand-alone intervention vs. incorporated in broader health care program? Intended for a particular patient population? Goals of the intervention, e.g., being more cost-effective to other interventions, replace or complement other solutions? (Note: Details about the intervention are provided in “Methods” under 5)

	1	2	3	4	5	
subitem not at all important	○	○	○	○	○	essential

Does your paper address subitem 2a-i? *

Copy and paste relevant sections from the manuscript (include quotes in quotation marks "like this" to indicate direct quotes from your manuscript), or elaborate on this item by providing additional information not in the ms, or briefly explain why the item is not applicable/relevant for your study

Yes, like this “On the basis of routine care, we have established a smartphone-based, family-physician collaborative mechanism for out-of-hospital jaundice screening mechanism. One randomized controlled trial (RCT) was conducted to evaluate whether this smartphone-based intervention could affect the relevant 30-day neonatal readmission and maternal anxiety compared with the routine care.”

2a-ii) Scientific background, rationale: What is known about the (type of) system

Scientific background, rationale: What is known about the (type of) system that is the object of the study (be sure to discuss the use of similar systems for other conditions/diagnoses, if appropiate), motivation for the study, i.e. what are the reasons for and what is the context for this specific study, from which stakeholder viewpoint is the study performed, potential impact of findings [2]. Briefly justify the choice of the comparator.

	1	2	3	4	5	
subitem not at all important	○	○	○	○	○	essential

Does your paper address subitem 2a-ii? *

Copy and paste relevant sections from the manuscript (include quotes in quotation marks "like this" to indicate direct quotes from your manuscript), or elaborate on this item by providing additional information not in the ms, or briefly explain why the item is not applicable/relevant for your study

Yes, like this “Mobile communication devices have been widely used in the healthcare sector. And such emerging information technologies may help overcome the barriers existing in routine monitoring of neonatal jaundice. Several clinical trials have found that smartphone-based jaundice testing was accurate and cost-effective in identifying neonates with high levels of TSB” “On the basis of routine care, we have established a smartphone-based, family-physician collaborative mechanism for out-of-hospital jaundice screening mechanism. One randomized controlled trial (RCT) was conducted to evaluate whether this smartphone-based intervention could affect the relevant 30-day neonatal readmission and maternal anxiety compared with the routine care.”

2b) In INTRODUCTION: Specific objectives or hypotheses

Does your paper address CONSORT subitem 2b? *

Copy and paste relevant sections from the manuscript (include quotes in quotation marks "like this" to indicate direct quotes from your manuscript), or elaborate on this item by providing additional information not in the ms, or briefly explain why the item is not applicable/relevant for your study

Yes., like this “One randomized controlled trial (RCT) was conducted to evaluate whether this smartphone-based intervention could affect the relevant 30-day neonatal readmission and maternal anxiety compared with the routine care.”

METHODS

3a) Description of trial design (such as parallel, factorial) including allocation ratio

Does your paper address CONSORT subitem 3a? *

Copy and paste relevant sections from the manuscript (include quotes in quotation marks "like this" to indicate direct quotes from your manuscript), or elaborate on this item by providing additional information not in the ms, or briefly explain why the item is not applicable/relevant for your study

“This study was an unblinded, parallel-assignment RCT conducted at three public hospitals in Hainan Province of China, from August 2019 to September 2020. Participants were randomly assigned to either the smartphone-based intervention group or the routine-care control group, and were followed up for 30 days.”

3b) Important changes to methods after trial commencement (such as eligibility criteria), with reasons

Does your paper address CONSORT subitem 3b? *

Copy and paste relevant sections from the manuscript (include quotes in quotation marks "like this" to indicate direct quotes from your manuscript), or elaborate on this item by providing additional information not in the ms, or briefly explain why the item is not applicable/relevant for your study

No. There is no change on methods after trial commencement.

3b-i) Bug fixes, Downtimes, Content Changes

Bug fixes, Downtimes, Content Changes: ehealth systems are often dynamic systems. A description of changes to methods therefore also includes important changes made on the intervention or comparator during the trial (e.g., major bug fixes or changes in the functionality or content) (5-iii) and other “unexpected events” that may have influenced study design such as staff changes, system failures/downtimes, etc. [2].

	1	2	3	4	5	
subitem not at all important	○	○	○	○	○	essential

Does your paper address subitem 3b-i?

Copy and paste relevant sections from the manuscript (include quotes in quotation marks "like this" to indicate direct quotes from your manuscript), or elaborate on this item by providing additional information not in the ms, or briefly explain why the item is not applicable/relevant for your study

No. There was no system change after trial commenced.

4a) Eligibility criteria for participants

Does your paper address CONSORT subitem 4a? *

Copy and paste relevant sections from the manuscript (include quotes in quotation marks "like this" to indicate direct quotes from your manuscript), or elaborate on this item by providing additional information not in the ms, or briefly explain why the item is not applicable/relevant for your study

Yes, like this "Neonates born in Maternal and Child Health Hospital of Hainan Province, Wanning People's Hospital and Chengmai People's Hospital and their mothers were recruited as participants. All mother-infant dyads met the hospital discharge criteria as judged by the bedside doctor, and they were about to be discharged home. Eligible mother was at least 16 years old with clear understanding and communication skills, owned a smartphone and was able to use it. Exclusion criteria included multiple births (e.g. twins), neonate who suffered from congenital disease, perinatal asphyxia, neonatal infection, hemolytic disease (positive Coombs' test) or other serious organic damage, family not living in Hainan Province within the study period."

4a-i) Computer / Internet literacy

Computer / Internet literacy is often an implicit "de facto" eligibility criterion - this should be explicitly clarified.

	1	2	3	4	5	
subitem not at all important	○	○	○	○	○	essential

Does your paper address subitem 4a-i?

Copy and paste relevant sections from the manuscript (include quotes in quotation marks "like this" to indicate direct quotes from your manuscript), or elaborate on this item by providing additional information not in the ms, or briefly explain why the item is not applicable/relevant for your study

Yes, like this "Eligible mother was at least 16 years old with clear understanding and communication skills, owned a smartphone and was able to use it."

4a-ii) Open vs. closed, web-based vs. face-to-face assessments:

Open vs. closed, web-based vs. face-to-face assessments: Mention how participants were recruited (online vs. offline), e.g., from an open access website or from a clinic, and clarify if this was a purely web-based trial, or there were face-to-face components (as part of the intervention or for assessment), i.e., to what degree got the study team to know the participant. In online-only trials, clarify if participants were quasi-anonymous and whether having multiple identities was possible or whether technical or logistical measures (e.g., cookies, email confirmation, phone calls) were used to detect/prevent these.

	1	2	3	4	5	
subitem not at all important	○	○	○	○	○	essential

Does your paper address subitem 4a-ii? *

Copy and paste relevant sections from the manuscript (include quotes in quotation marks "like this" to indicate direct quotes from your manuscript), or elaborate on this item by providing additional information not in the ms, or briefly explain why the item is not applicable/relevant for your study

Yes, like this “Qualified mother-infant dyads were identified and screened face to face by trained obstetric nurses prior to discharge from hospital.”

4a-iii) Information giving during recruitment

Information given during recruitment. Specify how participants were briefed for recruitment and in the informed consent procedures (e.g., publish the informed consent documentation as appendix, see also item X26), as this information may have an effect on user self-selection, user expectation and may also bias results.

	1	2	3	4	5	
subitem not at all important	○	○	○	○	○	essential

Does your paper address subitem 4a-iii?

Copy and paste relevant sections from the manuscript (include quotes in quotation marks "like this" to indicate direct quotes from your manuscript), or elaborate on this item by providing additional information not in the ms, or briefly explain why the item is not applicable/relevant for your study

Yes, like this "Qualified mother-infant dyads were identified and screened face to face by trained obstetric nurses prior to discharge from hospital. All enrolled samples were aware of the trial objective and process, written informed consent was obtained from mothers at study entry."

4b) Settings and locations where the data were collected

Does your paper address CONSORT subitem 4b? *

Copy and paste relevant sections from the manuscript (include quotes in quotation marks "like this" to indicate direct quotes from your manuscript), or elaborate on this item by providing additional information not in the ms, or briefly explain why the item is not applicable/relevant for your study

Yes, like this "On the 31st day after mother-infant dyad discharged from hospital, the maternal and neonatal outcomes were collected by trained obstetric nurses through telephone follow-up, and the last questionnaire survey was completed in September 2020. "
"At recruitment, maternal and neonatal basic demographic characteristics were collected through a self-assessed questionnaire. "

4b-i) Report if outcomes were (self-)assessed through online questionnaires

Clearly report if outcomes were (self-)assessed through online questionnaires (as common in web-based trials) or otherwise.

	1	2	3	4	5	
subitem not at all important	○	○	○	○	○	essential

Does your paper address subitem 4b-i? *

Copy and paste relevant sections from the manuscript (include quotes in quotation marks "like this" to indicate direct quotes from your manuscript), or elaborate on this item by providing additional information not in the ms, or briefly explain why the item is not applicable/relevant for your study

Yes, like this "On the 31st day after mother-infant dyad discharged from hospital, the maternal and neonatal outcomes were collected by trained obstetric nurses through telephone follow-up, and the last questionnaire survey was completed in September 2020." "Mothers were asked to answer: "Has your baby gone to the hospital again because of jaundice in the past 30 days?", and the corresponding options were Yes or No." " Secondary outcome was the maternal anxiety score associated with neonatal jaundice, which was measured by a self-designed scale (Multimedia Appendix 1). The scale consisted of 10 items with 4-level scoring method, and from "never happened" to "always happened", the scores were coded with 1 to 4 points respectively. The higher the total score (range: 10-40 points), the more serious the anxiety. It has good internal consistency with Cronbach's alpha=0.91. Questions were answered by mothers whose neonate developed jaundice after discharge home. " "At recruitment, maternal and neonatal basic demographic characteristics were collected through a self-assessed questionnaire. "

4b-ii) Report how institutional affiliations are displayed

Report how institutional affiliations are displayed to potential participants [on ehealth media], as affiliations with prestigious hospitals or universities may affect volunteer rates, use, and reactions with regards to an intervention.(Not a required item – describe only if this may bias results)

	1	2	3	4	5	
subitem not at all important	○	○	○	○	○	essential

Does your paper address subitem 4b-ii?

Copy and paste relevant sections from the manuscript (include quotes in quotation marks "like this" to indicate direct quotes from your manuscript), or elaborate on this item by providing additional information not in the ms, or briefly explain why the item is not applicable/relevant for your study

Not applicable. "From August 2019 to August 2020, mother-infant dyads were recruited on site from three public hospitals in Hainan Province, China." Participants were recruited directly.

5) The interventions for each group with sufficient details to allow replication, including how and when they were actually administered

5-i) Mention names, credential, affiliations of the developers, sponsors, and owners

Mention names, credential, affiliations of the developers, sponsors, and owners [6] (if authors/evaluators are owners or developer of the software, this needs to be declared in a “Conflict of interest” section or mentioned elsewhere in the manuscript).

	1	2	3	4	5	
subitem not at all important	○	○	○	○	○	essential

Does your paper address subitem 5-i?

Copy and paste relevant sections from the manuscript (include quotes in quotation marks "like this" to indicate direct quotes from your manuscript), or elaborate on this item by providing additional information not in the ms, or briefly explain why the item is not applicable/relevant for your study

Yes, like this “This work was supported by National Natural Science Foundation of China [grant numbers 71663016], Hainan Province Clinical Medical Center and The Excellent Talent Team of Hainan Province [No.QRCBT202121]. The funder had no role in the design and conduct of the study; collection, management, analysis, and interpretation of the data; preparation, review, or approval of the manuscript; and decision to submit the manuscript for publication.” "Yan, Yin and Luo are co-first authors. Fan, Yin, Gong, Luo and Yan conceptualized and designed the study. Luo, Yang, Wang, Feng, Xing, Y. Huang, C. Huang and Fan collected the data, which was supervised by Fan, Gong. Yan, Luo and Gong analyzed and interpreted the data. Yan and Yin drafted the article. All authors revised the manuscript for important intellectual content and approved the final article"

5-ii) Describe the history/development process

Describe the history/development process of the application and previous formative evaluations (e.g., focus groups, usability testing), as these will have an impact on adoption/use rates and help with interpreting results.

	1	2	3	4	5	
subitem not at all important	○	○	○	○	○	essential

Does your paper address subitem 5-ii?

Copy and paste relevant sections from the manuscript (include quotes in quotation marks "like this" to indicate direct quotes from your manuscript), or elaborate on this item by providing additional information not in the ms, or briefly explain why the item is not applicable/relevant for your study

Yes, like this “The Jaundice Mobile Monitoring App used in this study is a publicly available, free downloadable software in China that allows remote, non-invasive, and self-service jaundice monitoring and early warning. It is used in combination with a jaundice colorimetric card which is required to place on the chest of the child. After judging the image quality, light environment, and skin area, the App will automatically scan and take photos to obtain a clear image of the newborn skin. Once the cloud server receives the image data, jaundice value will be automatically calculated and displayed (automated image-based bilirubin test, AIB), along with an indication of the risk level of neonatal jaundice (Low risk: 2.6~10.1mg/L; medium risk: 10.2~17.1mg/L; high risk: ≥17.2mg/L). A clinical trial conducted to evaluate the screening accuracy of this App found strong concordance between AIB and TSB (r=0.97)”

5-iii) Revisions and updating

Revisions and updating. Clearly mention the date and/or version number of the application/intervention (and comparator, if applicable) evaluated, or describe whether the intervention underwent major changes during the evaluation process, or whether the development and/or content was “frozen” during the trial. Describe dynamic components such as news feeds or changing content which may have an impact on the replicability of the intervention (for unexpected events see item 3b).

	1	2	3	4	5	
subitem not at all important	○	○	○	○	○	essential

Does your paper address subitem 5-iii?

Copy and paste relevant sections from the manuscript (include quotes in quotation marks "like this" to indicate direct quotes from your manuscript), or elaborate on this item by providing additional information not in the ms, or briefly explain why the item is not applicable/relevant for your study

No major change was made after the App was launched.

5-iv) Quality assurance methods

Provide information on quality assurance methods to ensure accuracy and quality of information provided [1], if applicable.

	1	2	3	4	5	
subitem not at all important	○	○	○	○	○	essential

Does your paper address subitem 5-iv?

Copy and paste relevant sections from the manuscript (include quotes in quotation marks "like this" to indicate direct quotes from your manuscript), or elaborate on this item by providing additional information not in the ms, or briefly explain why the item is not applicable/relevant for your study

Yes, like this “A clinical trial conducted to evaluate the screening accuracy of this App found strong concordance between AIB and TSB (r=0.97)”

5-v) Ensure replicability by publishing the source code, and/or providing screenshots/screen-capture video, and/or providing flowcharts of the algorithms used

Ensure replicability by publishing the source code, and/or providing screenshots/screen-capture video, and/or providing flowcharts of the algorithms used. Replicability (i.e., other researchers should in principle be able to replicate the study) is a hallmark of scientific reporting.

	1	2	3	4	5	
subitem not at all important	○	○	○	○	○	essential

Does your paper address subitem 5-v?

Copy and paste relevant sections from the manuscript (include quotes in quotation marks "like this" to indicate direct quotes from your manuscript), or elaborate on this item by providing additional information not in the ms, or briefly explain why the item is not applicable/relevant for your study

The source code can be acquired by contacting author.

5-vi) Digital preservation

Digital preservation: Provide the URL of the application, but as the intervention is likely to change or disappear over the course of the years; also make sure the intervention is archived (Internet Archive, webcitation.org, and/or publishing the source code or screenshots/videos alongside the article). As pages behind login screens cannot be archived, consider creating demo pages which are accessible without login.

	1	2	3	4	5	
subitem not at all important	○	○	○	○	○	essential

Does your paper address subitem 5-vi?

Copy and paste relevant sections from the manuscript (include quotes in quotation marks "like this" to indicate direct quotes from your manuscript), or elaborate on this item by providing additional information not in the ms, or briefly explain why the item is not applicable/relevant for your study

您的回答

5-vii) Access

Access: Describe how participants accessed the application, in what setting/context, if they had to pay (or were paid) or not, whether they had to be a member of specific group. If known, describe how participants obtained “access to the platform and Internet” [1]. To ensure access for editors/reviewers/readers, consider to provide a “backdoor” login account or demo mode for reviewers/readers to explore the application (also important for archiving purposes, see vi).

	1	2	3	4	5	
subitem not at all important	○	○	○	○	○	essential

Does your paper address subitem 5-vii? *

Copy and paste relevant sections from the manuscript (include quotes in quotation marks "like this" to indicate direct quotes from your manuscript), or elaborate on this item by providing additional information not in the ms, or briefly explain why the item is not applicable/relevant for your study

Yes, like this “The Jaundice Mobile Monitoring App used in this study is a publicly available, free downloadable software in China that allows remote, non-invasive, and self-service jaundice monitoring and early warning.”

5-viii) Mode of delivery, features/functionalities/components of the intervention and comparator, and the theoretical framework

Describe mode of delivery, features/functionalities/components of the intervention and comparator, and the theoretical framework [6] used to design them (instructional strategy [1], behaviour change techniques, persuasive features, etc., see e.g., [7, 8] for terminology). This includes an in-depth description of the content (including where it is coming from and who developed it) [1],” whether [and how] it is tailored to individual circumstances and allows users to track their progress and receive feedback” [6]. This also includes a description of communication delivery channels and – if computer-mediated communication is a component – whether communication was synchronous or asynchronous [6]. It also includes information on presentation strategies [1], including page design principles, average amount of text on pages, presence of hyperlinks to other resources, etc. [1].

	1	2	3	4	5	
subitem not at all important	○	○	○	○	○	essential

Does your paper address subitem 5-viii? *

Copy and paste relevant sections from the manuscript (include quotes in quotation marks "like this" to indicate direct quotes from your manuscript), or elaborate on this item by providing additional information not in the ms, or briefly explain why the item is not applicable/relevant for your study

Yes, like this “The Jaundice Mobile Monitoring App used in this study is a publicly available, free downloadable software in China that allows remote, non-invasive, and self-service jaundice monitoring and early warning.”

5-ix) Describe use parameters

Describe use parameters (e.g., intended “doses” and optimal timing for use). Clarify what instructions or recommendations were given to the user, e.g., regarding timing, frequency, heaviness of use, if any, or was the intervention used ad libitum.

	1	2	3	4	5	
subitem not at all important	○	○	○	○	○	essential

Does your paper address subitem 5-ix?

Copy and paste relevant sections from the manuscript (include quotes in quotation marks "like this" to indicate direct quotes from your manuscript), or elaborate on this item by providing additional information not in the ms, or briefly explain why the item is not applicable/relevant for your study

Yes, like this “The Jaundice Mobile Monitoring App used in this study is a publicly available, free downloadable software in China that allows remote, non-invasive, and self-service jaundice monitoring and early warning. It is used in combination with a jaundice colorimetric card which is required to place on the chest of the child. After judging the image quality, light environment, and skin area, the App will automatically scan and take photos to obtain a clear image of the newborn skin. Once the cloud server receives the image data, jaundice value will be automatically calculated and displayed (automated image-based bilirubin test, AIB), along with an indication of the risk level of neonatal jaundice (Low risk: 2.6~10.1mg/L; medium risk: 10.2~17.1mg/L; high risk: ≥17.2mg/L). A clinical trial conducted to evaluate the screening accuracy of this App found strong concordance between AIB and TSB (r=0.97)” "Intervention group mothers received free appropriative jaundice colorimetric cards and installed the Jaundice Mobile Monitoring App on their smartphones at hospital discharge. And they were instructed by trained obstetric nurses about data uploading method. Meanwhile, they were required to detect neonatal bilirubin values using the App when needed after returning home. We assigned a paediatrician from the hospital where the delivery took place to each mother, and bound the mother’s and the doctor's versions of Jaundice Mobile Monitoring App together, both parties could use the App platform for communication and consultation. Paediatricians would be able to access the dynamic changes and risk prompts of neonatal bilirubin values synchronously, so as to judge the infants’ situation and guide the mother to take correct actions. If neonatal bilirubin was considered to be at high risk level or the condition was quite severe, the paediatrician would alert mother to bring the child return to hospital for further examination and specialist treatment. In addition, paediatricians would supervise and remind mothers to measure newborn infants’ jaundice level every day through the App."

5-x) Clarify the level of human involvement

Clarify the level of human involvement (care providers or health professionals, also technical assistance) in the e-intervention or as co-intervention (detail number and expertise of professionals involved, if any, as well as “type of assistance offered, the timing and frequency of the support, how it is initiated, and the medium by which the assistance is delivered”. It may be necessary to distinguish between the level of human involvement required for the trial, and the level of human involvement required for a routine application outside of a RCT setting (discuss under item 21 – generalizability).

	1	2	3	4	5	
subitem not at all important	○	○	○	○	○	essential

Does your paper address subitem 5-x?

Copy and paste relevant sections from the manuscript (include quotes in quotation marks "like this" to indicate direct quotes from your manuscript), or elaborate on this item by providing additional information not in the ms, or briefly explain why the item is not applicable/relevant for your study

Yes, like this "Intervention group mothers received free appropriative jaundice colorimetric cards and installed the Jaundice Mobile Monitoring App on their smartphones at hospital discharge. And they were instructed by trained obstetric nurses about data uploading method. Meanwhile, they were required to detect neonatal bilirubin values using the App when needed after returning home. We assigned a paediatrician from the hospital where the delivery took place to each mother, and bound the mother's and the doctor's versions of Jaundice Mobile Monitoring App together, both parties could use the App platform for communication and consultation. Paediatricians would be able to access the dynamic changes and risk prompts of neonatal bilirubin values synchronously, so as to judge the infants' situation and guide the mother to take correct actions. If neonatal bilirubin was considered to be at high risk level or the condition was quite severe, the paediatrician would alert mother to bring the child return to hospital for further examination and specialist treatment. In addition, paediatricians would supervise and remind mothers to measure newborn infants' jaundice level every day through the App."

5-xi) Report any prompts/reminders used

Report any prompts/reminders used: Clarify if there were prompts (letters, emails, phone calls, SMS) to use the application, what triggered them, frequency etc. It may be necessary to distinguish between the level of prompts/reminders required for the trial, and the level of prompts/reminders for a routine application outside of a RCT setting (discuss under item 21 – generalizability).

	1	2	3	4	5	
subitem not at all important	○	○	○	○	○	essential

Does your paper address subitem 5-xi? *

Copy and paste relevant sections from the manuscript (include quotes in quotation marks "like this" to indicate direct quotes from your manuscript), or elaborate on this item by providing additional information not in the ms, or briefly explain why the item is not applicable/relevant for your study

Yes, like this “We assigned a paediatrician from the hospital where the delivery took place to each mother, and bound the mother’s and the doctor's versions of Jaundice Mobile Monitoring App together, both parties could use the App platform for communication and consultation.”

5-xii) Describe any co-interventions (incl. training/support)

Describe any co-interventions (incl. training/support): Clearly state any interventions that are provided in addition to the targeted eHealth intervention, as ehealth intervention may not be designed as stand-alone intervention. This includes training sessions and support [1]. It may be necessary to distinguish between the level of training required for the trial, and the level of training for a routine application outside of a RCT setting (discuss under item 21 – generalizability.

	1	2	3	4	5	
subitem not at all important	○	○	○	○	○	essential

Does your paper address subitem 5-xii? *

Copy and paste relevant sections from the manuscript (include quotes in quotation marks "like this" to indicate direct quotes from your manuscript), or elaborate on this item by providing additional information not in the ms, or briefly explain why the item is not applicable/relevant for your study

Yes, like this "Participants randomized to the control group received routine care. Under the National Basic Public Health Service Program in China[26], the primary health care institutions conduct newborn home visits (within 1 week of hospital discharge) and full-term health management for newborns (28-30 days after birth). Neonates with high risk factors such as low birth weight and prematurity are visited more frequently according to the actual situation. " "Participants randomized to the intervention group received routine care and the smartphone-based, family-physician collaborative neonatal jaundice screening mechanism. That is, under the remote guidance of a paediatrician, mother monitored routinely neonatal jaundice at home using a smartphone App. "

6a) Completely defined pre-specified primary and secondary outcome measures, including how and when they were assessed

Does your paper address CONSORT subitem 6a? *

Copy and paste relevant sections from the manuscript (include quotes in quotation marks "like this" to indicate direct quotes from your manuscript), or elaborate on this item by providing additional information not in the ms, or briefly explain why the item is not applicable/relevant for your study

Yes, like this "On the 31st day after mother-infant dyad discharged from hospital, the maternal and neonatal outcomes were collected by trained obstetric nurses through telephone follow-up, and the last questionnaire survey was completed in September 2020. The primary outcome was the neonatal readmission rate due to jaundice, which was defined as the ratio of the number of neonates readmit to hospitals for jaundice within 30 days of first discharge to the total number of neonates in each group. Mothers were asked to answer: "Has your baby gone to the hospital again because of jaundice in the past 30 days?", and the corresponding options were Yes or No. Secondary outcome was the maternal anxiety score associated with neonatal jaundice, which was measured by a self-designed scale (Multimedia Appendix 1). The scale consisted of 10 items with 4-level scoring method, and from "never happened" to "always happened", the scores were coded with 1 to 4 points respectively. The higher the total score (range: 10-40 points), the more serious the anxiety. It has good internal consistency with Cronbach's alpha=0.91. Questions were answered by mothers whose neonate developed jaundice after discharge home. In addition, the intervention group mothers were also asked to rate the Jaundice Mobile Monitoring AppAPP in five aspects (convenience, trustworthiness, recommendation, generalizability and satisfaction), so as to assess the acceptability of the AppAPP. "

6a-i) Online questionnaires: describe if they were validated for online use and apply CHERRIES items to describe how the questionnaires were designed/deployed

If outcomes were obtained through online questionnaires, describe if they were validated for online use and apply CHERRIES items to describe how the questionnaires were designed/deployed [9].

	1	2	3	4	5	
subitem not at all important	○	○	○	○	○	essential

Does your paper address subitem 6a-i?

Copy and paste relevant sections from manuscript text

Yes, like this "On the 31st day after mother-infant dyad discharged from hospital, the maternal and neonatal outcomes were collected by trained obstetric nurses through telephone follow-up, and the last questionnaire survey was completed in September 2020. " "Secondary outcome was the maternal anxiety score associated with neonatal jaundice, which was measured by a self-designed scale (Multimedia Appendix 1). The scale consisted of 10 items with 4-level scoring method, and from "never happened" to "always happened", the scores were coded with 1 to 4 points respectively. The higher the total score (range: 10-40 points), the more serious the anxiety. It has good internal consistency with Cronbach's alpha=0.91. "

6a-ii) Describe whether and how “use” (including intensity of use/dosage) was defined/measured/monitored

Describe whether and how “use” (including intensity of use/dosage) was defined/measured/monitored (logins, logfile analysis, etc.). Use/adoption metrics are important process outcomes that should be reported in any ehealth trial.

	1	2	3	4	5	
subitem not at all important	○	○	○	○	○	essential

Does your paper address subitem 6a-ii?

Copy and paste relevant sections from manuscript text

Yes, like this “Meanwhile, they were required to detect neonatal bilirubin values using the App when needed after returning home.”

6a-iii) Describe whether, how, and when qualitative feedback from participants was obtained

Describe whether, how, and when qualitative feedback from participants was obtained (e.g., through emails, feedback forms, interviews, focus groups).

	1	2	3	4	5	
subitem not at all important	○	○	○	○	○	essential

Does your paper address subitem 6a-iii?

Copy and paste relevant sections from manuscript text

Yes, like this “On the 31st day after mother-infant dyad discharged from hospital, the maternal and neonatal outcomes were collected by trained obstetric nurses through telephone follow-up”

6b) Any changes to trial outcomes after the trial commenced, with reasons

Does your paper address CONSORT subitem 6b? *

Copy and paste relevant sections from the manuscript (include quotes in quotation marks "like this" to indicate direct quotes from your manuscript), or elaborate on this item by providing additional information not in the ms, or briefly explain why the item is not applicable/relevant for your study

No change to trial outcomes after the trial commenced.

7a) How sample size was determined

NPT: When applicable, details of whether and how the clustering by care provides or centers was addressed

7a-i) Describe whether and how expected attrition was taken into account when calculating the sample size

Describe whether and how expected attrition was taken into account when calculating the sample size.

	1	2	3	4	5	
subitem not at all important	○	○	○	○	○	essential

Does your paper address subitem 7a-i?

Copy and paste relevant sections from manuscript title (include quotes in quotation marks "like this" to indicate direct quotes from your manuscript), or elaborate on this item by providing additional information not in the ms, or briefly explain why the item is not applicable/relevant for your study

Yes, like this "A sample size calculation was preformed based on primary outcome. Based on previous studies and our hypothesis that the 30-day neonatal readmission rate due to jaundice would relatively reduce 50% in this trial, as well as considered about 10% attrition rate, we estimated that about 600 neonates were needed for each group to ensure the difference could be detected at 80% power (α= .05, 2-sided), i.e., a total requirement of 1200 mother-infant dyads at least."

7b) When applicable, explanation of any interim analyses and stopping guidelines

Does your paper address CONSORT subitem 7b? *

Copy and paste relevant sections from the manuscript (include quotes in quotation marks "like this" to indicate direct quotes from your manuscript), or elaborate on this item by providing additional information not in the ms, or briefly explain why the item is not applicable/relevant for your study

Not applicable. So we did not provide the explanation of any interim analyses and stopping guidelines.

8a) Method used to generate the random allocation sequence

NPT: When applicable, how care providers were allocated to each trial group

Does your paper address CONSORT subitem 8a? *

Copy and paste relevant sections from the manuscript (include quotes in quotation marks "like this" to indicate direct quotes from your manuscript), or elaborate on this item by providing additional information not in the ms, or briefly explain why the item is not applicable/relevant for your study

Yes, like this “After baseline information were collected, the trained obstetric nurses carried out simple randomization (draw of lots), and allocated each mother-infant dyad to the intervention and control groups.”

8b) Type of randomisation; details of any restriction (such as blocking and block size)

Does your paper address CONSORT subitem 8b? *

Copy and paste relevant sections from the manuscript (include quotes in quotation marks "like this" to indicate direct quotes from your manuscript), or elaborate on this item by providing additional information not in the ms, or briefly explain why the item is not applicable/relevant for your study

Yes, like this "simple randomization (draw of lots)"

9) Mechanism used to implement the random allocation sequence (such as sequentially numbered containers), describing any steps taken to conceal the sequence until interventions were assigned

Does your paper address CONSORT subitem 9? *

Copy and paste relevant sections from the manuscript (include quotes in quotation marks "like this" to indicate direct quotes from your manuscript), or elaborate on this item by providing additional information not in the ms, or briefly explain why the item is not applicable/relevant for your study

Yes, like this “After baseline information were collected, the trained obstetric nurses carried out simple randomization (draw of lots), and allocated each mother-infant dyad to the intervention and control groups.”

10) Who generated the random allocation sequence, who enrolled participants, and who assigned participants to interventions

Does your paper address CONSORT subitem 10? *

Copy and paste relevant sections from the manuscript (include quotes in quotation marks "like this" to indicate direct quotes from your manuscript), or elaborate on this item by providing additional information not in the ms, or briefly explain why the item is not applicable/relevant for your study

Yes, like this "Qualified mother-infant dyads were identified and screened face to face by trained obstetric nurses prior to discharge from hospital" "After baseline information were collected, the trained obstetric nurses carried out simple randomization (draw of lots), and allocated each mother-infant dyad to the intervention and control groups."

11a) If done, who was blinded after assignment to interventions (for example, participants, care providers, those assessing outcomes) and how

NPT: Whether or not administering co-interventions were blinded to group assignment

11a-i) Specify who was blinded, and who wasn't

Specify who was blinded, and who wasn't. Usually, in web-based trials it is not possible to blind the participants [1, 3] (this should be clearly acknowledged), but it may be possible to blind outcome assessors, those doing data analysis or those administering co-interventions (if any).

	1	2	3	4	5	
subitem not at all important	○	○	○	○	○	essential

Does your paper address subitem 11a-i? *

Copy and paste relevant sections from the manuscript (include quotes in quotation marks "like this" to indicate direct quotes from your manuscript), or elaborate on this item by providing additional information not in the ms, or briefly explain why the item is not applicable/relevant for your study

Yes, like this "This study was an unblinded, parallel-assignment RCT" "Because of the nature of the intervention, it was not possible to blind participants, nor the pediatric nurses who were responsible for participant recruitment and follow-up"

11a-ii) Discuss e.g., whether participants knew which intervention was the "intervention of interest" and which one was the "comparator"

Informed consent procedures (4a-ii) can create biases and certain expectations - discuss e.g., whether participants knew which intervention was the "intervention of interest" and which one was the "comparator".

	1	2	3	4	5	
subitem not at all important	○	○	○	○	○	essential

Does your paper address subitem 11a-ii?

Copy and paste relevant sections from the manuscript (include quotes in quotation marks "like this" to indicate direct quotes from your manuscript), or elaborate on this item by providing additional information not in the ms, or briefly explain why the item is not applicable/relevant for your study

Yes. participants knew which intervention was the "intervention of interest" and which one was the "control"

11b) If relevant, description of the similarity of interventions

(this item is usually not relevant for ehealth trials as it refers to similarity of a placebo or sham intervention to a active medication/intervention)

Does your paper address CONSORT subitem 11b? *

Copy and paste relevant sections from the manuscript (include quotes in quotation marks "like this" to indicate direct quotes from your manuscript), or elaborate on this item by providing additional information not in the ms, or briefly explain why the item is not applicable/relevant for your study

Yes, like this "Participants randomized to the control group received routine care. Under the National Basic Public Health Service Program in China[26], the primary health care institutions conduct newborn home visits (within 1 week of hospital discharge) and full-term health management for newborns (28-30 days after birth). Neonates with high risk factors such as low birth weight and prematurity are visited more frequently according to the actual situation. " "Participants randomized to the intervention group received routine care and the smartphone-based, family-physician collaborative neonatal jaundice screening mechanism. That is, under the remote guidance of a paediatrician, mother monitored routinely neonatal jaundice at home using a smartphone App."

12a) Statistical methods used to compare groups for primary and secondary outcomes

NPT: When applicable, details of whether and how the clustering by care providers or centers was addressed

Does your paper address CONSORT subitem 12a? *

Copy and paste relevant sections from the manuscript (include quotes in quotation marks "like this" to indicate direct quotes from your manuscript), or elaborate on this item by providing additional information not in the ms, or briefly explain why the item is not applicable/relevant for your study

Yes, like this "All analysis followed intention-to-treat principles, including all the randomly assigned mother-infant dyads. We examined the distributions of baseline characteristics using descriptive statistics, and Pearson chi-square test, Student t-test and Wilcoxon rank sum test were applied to compare the difference between two groups. We conducted analysis using a binary logistic regression model for primary outcome and a multiple linear regression model for secondary outcome, respectively, and reported the odds ratio (OR), regression coefficient (β) and 95% confidence intervals (CI). We adjusted all models for observed baseline maternal and neonatal characteristics (maternal age, nation, education level, employment status, medical insurance, residence, convenience of access to health care, economic status and neonatal gender, gestational age, birth weight, feeding patterns, parity, status of jaundice before discharge, delivery method).
We added some post hoc analysis. Firstly, based on least squares regression and identity link function, the generalized linear models were used to enable estimation of adjusted risk difference (RD) for binary outcome and adjusted mean difference (MD) for continuous outcome. Besides, we fitted a linear mixed-effects repeated measures model to maternal anxiety scores at baseline and follow-up, thus capturing whether there was greater improvement in the smartphone-based intervention group than in control group. Sensitivity analysis were done to assess the robustness of the primary and secondary outcomes analysis. We carried out multivariate imputation using chained equations to impute all missing data, including outcomes failure to followed up. "

12a-i) Imputation techniques to deal with attrition / missing values

Imputation techniques to deal with attrition / missing values: Not all participants will use the intervention/comparator as intended and attrition is typically high in ehealth trials. Specify how participants who did not use the application or dropped out from the trial were treated in the statistical analysis (a complete case analysis is strongly discouraged, and simple imputation techniques such as LOCF may also be problematic [4]).

	1	2	3	4	5	
subitem not at all important	○	○	○	○	○	essential

Does your paper address subitem 12a-i? *

Copy and paste relevant sections from the manuscript (include quotes in quotation marks "like this" to indicate direct quotes from your manuscript), or elaborate on this item by providing additional information not in the ms, or briefly explain why the item is not applicable/relevant for your study

Yes, like this “We carried out multivariate imputation using chained equations to impute all missing data, including outcomes failure to followed up.”

12b) Methods for additional analyses, such as subgroup analyses and adjusted analyses

Does your paper address CONSORT subitem 12b? *

Copy and paste relevant sections from the manuscript (include quotes in quotation marks "like this" to indicate direct quotes from your manuscript), or elaborate on this item by providing additional information not in the ms, or briefly explain why the item is not applicable/relevant for your study

Yes, like this "We adjusted all models for observed baseline maternal and neonatal characteristics (maternal age, nation, education level, employment status, medical insurance, residence, convenience of access to health care, economic status and neonatal gender, gestational age, birth weight, feeding patterns, parity, status of jaundice before discharge, delivery method)” “We added some post hoc analysis.” “Sensitivity analysis were done to assess the robustness of the primary and secondary outcomes analysis."

X26) REB/IRB Approval and Ethical Considerations [recommended as subheading under "Methods"] (not a CONSORT item)

X26-i) Comment on ethics committee approval

	1	2	3	4	5	
subitem not at all important	○	○	○	○	○	essential

Does your paper address subitem X26-i?

Copy and paste relevant sections from the manuscript (include quotes in quotation marks "like this" to indicate direct quotes from your manuscript), or elaborate on this item by providing additional information not in the ms, or briefly explain why the item is not applicable/relevant for your study

Yes, like this "The Ethics Committee of Maternal and Child Health Hospital of Hainan Province approved the trial protocol, and all participants provided written informed assent."

x26-ii) Outline informed consent procedures

Outline informed consent procedures e.g., if consent was obtained offline or online (how? Checkbox, etc.?), and what information was provided (see 4a-ii). See [6] for some items to be included in informed consent documents.

	1	2	3	4	5	
subitem not at all important	○	○	○	○	○	essential

Does your paper address subitem X26-ii?

Copy and paste relevant sections from the manuscript (include quotes in quotation marks "like this" to indicate direct quotes from your manuscript), or elaborate on this item by providing additional information not in the ms, or briefly explain why the item is not applicable/relevant for your study

Yes, like this "All enrolled samples were aware of the trial objective and process, written informed consent was obtained from mothers at study entry."

X26-iii) Safety and security procedures

Safety and security procedures, incl. privacy considerations, and any steps taken to reduce the likelihood or detection of harm (e.g., education and training, availability of a hotline)

	1	2	3	4	5	
subitem not at all important	○	○	○	○	○	essential

Does your paper address subitem X26-iii?

Copy and paste relevant sections from the manuscript (include quotes in quotation marks "like this" to indicate direct quotes from your manuscript), or elaborate on this item by providing additional information not in the ms, or briefly explain why the item is not applicable/relevant for your study

Yes, like this "Paediatricians would be able to access the dynamic changes and risk prompts of neonatal bilirubin values synchronously, so as to judge the infants' situation and guide the mother to take correct actions. If neonatal bilirubin was considered to be at high risk level or the condition was quite severe, the paediatrician would alert mother to bring the child return to hospital for further examination and specialist treatment."

RESULTS

13a) For each group, the numbers of participants who were randomly assigned, received intended treatment, and were analysed for the primary outcome

NPT: The number of care providers or centers performing the intervention in each group and the number of patients treated by each care provider in each center

Does your paper address CONSORT subitem 13a? *

Copy and paste relevant sections from the manuscript (include quotes in quotation marks "like this" to indicate direct quotes from your manuscript), or elaborate on this item by providing additional information not in the ms, or briefly explain why the item is not applicable/relevant for your study

Yes, like this "From August 2019 to August 2020, we screened 5635 mother-infant dyads in three public hospitals, and 1424 were eligible (Figure 1). Among them, 712 were randomly classified into the smartphone-based intervention group; 712 into routine-care control group. On the 31st day of intervention, 605 intervention group and 582 control group mother-infant dyads were followed up and available for analysis, others were lost because of refusal to follow up, drop-outs, etc., with a total retention rate of 83.4%. " "Figure 1"and "Table 3"

13b) For each group, losses and exclusions after randomisation, together with reasons

Does your paper address CONSORT subitem 13b? (NOTE: Preferably, this is shown in a CONSORT flow diagram) *

Copy and paste relevant sections from the manuscript (include quotes in quotation marks "like this" to indicate direct quotes from your manuscript), or elaborate on this item by providing additional information not in the ms, or briefly explain why the item is not applicable/relevant for your study

Yes, like this " On the 31st day of intervention, 605 intervention group and 582 control group mother-infant dyads were followed up and available for analysis, others were lost because of refusal to follow up, drop-outs, etc., with a total retention rate of 83.4%. " “Figure 1”

13b-i) Attrition diagram

Strongly recommended: An attrition diagram (e.g., proportion of participants still logging in or using the intervention/comparator in each group plotted over time, similar to a survival curve) or other figures or tables demonstrating usage/dose/engagement.

	1	2	3	4	5	
subitem not at all important	○	○	○	○	○	essential

Does your paper address subitem 13b-i?

Copy and paste relevant sections from the manuscript or cite the figure number if applicable (include quotes in quotation marks "like this" to indicate direct quotes from your manuscript), or elaborate on this item by providing additional information not in the ms, or briefly explain why the item is not applicable/relevant for your study

您的回答

14a) Dates defining the periods of recruitment and follow-up

Does your paper address CONSORT subitem 14a? *

Copy and paste relevant sections from the manuscript (include quotes in quotation marks "like this" to indicate direct quotes from your manuscript), or elaborate on this item by providing additional information not in the ms, or briefly explain why the item is not applicable/relevant for your study

Yes， like this “From August 2019 to August 2020, we screened 5635 mother-infant dyads in three public hospitals, and 1424 were eligible” "On the 31st day of intervention, 605 intervention group and 582 control group mother-infant dyads were followed up and available for analysis"

14a-i) Indicate if critical “secular events” fell into the study period

Indicate if critical “secular events” fell into the study period, e.g., significant changes in Internet resources available or “changes in computer hardware or Internet delivery resources”

	1	2	3	4	5	
subitem not at all important	○	○	○	○	○	essential

Does your paper address subitem 14a-i?

Copy and paste relevant sections from the manuscript (include quotes in quotation marks "like this" to indicate direct quotes from your manuscript), or elaborate on this item by providing additional information not in the ms, or briefly explain why the item is not applicable/relevant for your study

No critical “secular events” fell into the study period.

14b) Why the trial ended or was stopped (early)

Does your paper address CONSORT subitem 14b? *

Copy and paste relevant sections from the manuscript (include quotes in quotation marks "like this" to indicate direct quotes from your manuscript), or elaborate on this item by providing additional information not in the ms, or briefly explain why the item is not applicable/relevant for your study

Not applicable.

15) A table showing baseline demographic and clinical characteristics for each group

NPT: When applicable, a description of care providers (case volume, qualification, expertise, etc.) and centers (volume) in each group

Does your paper address CONSORT subitem 15? *

Copy and paste relevant sections from the manuscript (include quotes in quotation marks "like this" to indicate direct quotes from your manuscript), or elaborate on this item by providing additional information not in the ms, or briefly explain why the item is not applicable/relevant for your study

Yes, like this "Table 1 Baseline characteristics of mothers" "Table2 Baseline characteristics of neonates"

15-i) Report demographics associated with digital divide issues

In ehealth trials it is particularly important to report demographics associated with digital divide issues, such as age, education, gender, social-economic status, computer/Internet/ehealth literacy of the participants, if known.

	1	2	3	4	5	
subitem not at all important	○	○	○	○	○	essential

Does your paper address subitem 15-i? *

Copy and paste relevant sections from the manuscript (include quotes in quotation marks "like this" to indicate direct quotes from your manuscript), or elaborate on this item by providing additional information not in the ms, or briefly explain why the item is not applicable/relevant for your study

Yes, like this "The median age of mothers was 29 years old (IQR=26-32), 460 (32.3%) lived in rural areas, and the mean (SD) maternal anxiety score associated with neonatal jaundice was 19.6(6.3) (Table 1). 714 neonates were male (50.1%), 899 (63.1%) were natural delivery and most of them were breastfed 1062 (74.6%) (Table 2). Baseline characteristics were well balanced between the two groups. Moreover, similar demographic characteristics were observed between those who completed this trial and uncompleted (Multimedia Appendix 2). "

16) For each group, number of participants (denominator) included in each analysis and whether the analysis was by original assigned groups

16-i) Report multiple "denominators" and provide definitions

Report multiple "denominators" and provide definitions: Report N's (and effect sizes) "across a range of study participation [and use] thresholds" [1], e.g., N exposed, N consented, N used more than x times, N used more than y weeks, N participants "used" the intervention/comparator at specific pre-defined time points of interest (in absolute and relative numbers per group). Always clearly define "use" of the intervention.

	1	2	3	4	5	
subitem not at all important	○	○	○	○	○	essential

Does your paper address subitem 16-i? *

Copy and paste relevant sections from the manuscript (include quotes in quotation marks "like this" to indicate direct quotes from your manuscript), or elaborate on this item by providing additional information not in the ms, or briefly explain why the item is not applicable/relevant for your study

Yes, like this "Among them, 712 were randomly classified into the smartphone-based intervention group; 712 into routine-care control group. On the 31st day of intervention, 605 intervention group and 582 control group mother-infant dyads were followed up and available for analysis, others were lost because of refusal to follow up, drop-outs, etc., with a total retention rate of 83.4%."

16-ii) Primary analysis should be intent-to-treat

Primary analysis should be intent-to-treat, secondary analyses could include comparing only "users", with the appropriate caveats that this is no longer a randomized sample (see 18-i).

	1	2	3	4	5	
subitem not at all important	○	○	○	○	○	essential

Does your paper address subitem 16-ii?

Copy and paste relevant sections from the manuscript (include quotes in quotation marks "like this" to indicate direct quotes from your manuscript), or elaborate on this item by providing additional information not in the ms, or briefly explain why the item is not applicable/relevant for your study

Yes, like this "All analysis followed intention-to-treat principles, including all the randomly assigned mother-infant dyads"

17a) For each primary and secondary outcome, results for each group, and the estimated effect size and its precision (such as 95% confidence interval)

Does your paper address CONSORT subitem 17a? *

Copy and paste relevant sections from the manuscript (include quotes in quotation marks "like this" to indicate direct quotes from your manuscript), or elaborate on this item by providing additional information not in the ms, or briefly explain why the item is not applicable/relevant for your study

Yes, like this "Within 30 days of the first hospital discharge, 412 (68.1%) and 408 (70.1%) neonates in intervention and control groups who were successfully followed up appeared jaundice symptoms, respectively, with no statistical difference between the two groups. The smartphone-based intervention was significantly associated with a decrease (11.7 vs 24.2; RD, 12.5%; 95%CI， -16.8% to -8.2%) in the neonatal readmission rate due to jaundice (OR, 0.4; 95% CI, 0.3~0.6). After adjusting the model, our intervention remained observably effective in reducing the risk of readmission (OR, 0.4; 95% CI, 0.3~0.5), although the degree of reduction was smaller (RD, -10.5%; 95%CI, -15.9% to -5.0%) (Table 3)." "Maternal anxiety scores associated with neonatal jaundice were apparently lower in smartphone-based intervention group compared with the control group (MD, -3.7; 95% CI, -4.6 to -2.9). The adjusted model showed a similar intervention effect (MD, -3.6; 95% CI, -4.5 to -2.8) (Table 3).
"

17a-i) Presentation of process outcomes such as metrics of use and intensity of use

In addition to primary/secondary (clinical) outcomes, the presentation of process outcomes such as metrics of use and intensity of use (dose, exposure) and their operational definitions is critical. This does not only refer to metrics of attrition (13-b) (often a binary variable), but also to more continuous exposure metrics such as “average session length”. These must be accompanied by a technical description how a metric like a “session” is defined (e.g., timeout after idle time) [1] (report under item 6a).

	1	2	3	4	5	
subitem not at all important	○	○	○	○	○	essential

Does your paper address subitem 17a-i?

Copy and paste relevant sections from the manuscript (include quotes in quotation marks "like this" to indicate direct quotes from your manuscript), or elaborate on this item by providing additional information not in the ms, or briefly explain why the item is not applicable/relevant for your study

Not applicable.

17b) For binary outcomes, presentation of both absolute and relative effect sizes is recommended

Does your paper address CONSORT subitem 17b? *

Copy and paste relevant sections from the manuscript (include quotes in quotation marks "like this" to indicate direct quotes from your manuscript), or elaborate on this item by providing additional information not in the ms, or briefly explain why the item is not applicable/relevant for your study

Yes, like this "Within 30 days of the first hospital discharge, 412 (68.1%) and 408 (70.1%) neonates in intervention and control groups who were successfully followed up appeared jaundice symptoms, respectively, with no statistical difference between the two groups. The smartphone-based intervention was significantly associated with a decrease (11.7 vs 24.2; RD, 12.5%; 95%CI， -16.8% to -8.2%) in the neonatal readmission rate due to jaundice (OR, 0.4; 95% CI, 0.3~0.6). After adjusting the model, our intervention remained observably effective in reducing the risk of readmission (OR, 0.4; 95% CI, 0.3~0.5), although the degree of reduction was smaller (RD, -10.5%; 95%CI, -15.9% to -5.0%) (Table 3)."

18) Results of any other analyses performed, including subgroup analyses and adjusted analyses, distinguishing pre-specified from exploratory

Does your paper address CONSORT subitem 18? *

Copy and paste relevant sections from the manuscript (include quotes in quotation marks "like this" to indicate direct quotes from your manuscript), or elaborate on this item by providing additional information not in the ms, or briefly explain why the item is not applicable/relevant for your study

Yes, like this "We performed a post hoc analysis to examine whether improvement in maternal anxiety could be explained by underlying temporal trends. There were 347 intervention group and 348 control group neonates developed jaundice both at baseline and during intervention period follow-up. For these participants, maternal anxiety is shown in Figure 2. From baseline to 31st day follow-up, the mean maternal anxiety score of intervention group reduced by 4.14 (95%CI, -4.9-3.4), however, the control group slightly increased by 1.4 (95%CI, 0.5 to 2.4). Similarly, maternal anxiety scores were significantly lower in intervention group than control group at post-intervention follow-up (Adjusted difference, -4.2, 95% CI, -5.1 to -3.3). "

18-i) Subgroup analysis of comparing only users

A subgroup analysis of comparing only users is not uncommon in ehealth trials, but if done, it must be stressed that this is a self-selected sample and no longer an unbiased sample from a randomized trial (see 16-iii).

	1	2	3	4	5	
subitem not at all important	○	○	○	○	○	essential

Does your paper address subitem 18-i?

Copy and paste relevant sections from the manuscript (include quotes in quotation marks "like this" to indicate direct quotes from your manuscript), or elaborate on this item by providing additional information not in the ms, or briefly explain why the item is not applicable/relevant for your study

您的回答

19) All important harms or unintended effects in each group

(for specific guidance see CONSORT for harms)

Does your paper address CONSORT subitem 19? *

Copy and paste relevant sections from the manuscript (include quotes in quotation marks "like this" to indicate direct quotes from your manuscript), or elaborate on this item by providing additional information not in the ms, or briefly explain why the item is not applicable/relevant for your study

There were no harms or unintended effects in each group.

19-i) Include privacy breaches, technical problems

Include privacy breaches, technical problems. This does not only include physical “harm” to participants, but also incidents such as perceived or real privacy breaches [1], technical problems, and other unexpected/unintended incidents. “Unintended effects” also includes unintended positive effects [2].

	1	2	3	4	5	
subitem not at all important	○	○	○	○	○	essential

Does your paper address subitem 19-i?

Copy and paste relevant sections from the manuscript (include quotes in quotation marks "like this" to indicate direct quotes from your manuscript), or elaborate on this item by providing additional information not in the ms, or briefly explain why the item is not applicable/relevant for your study

There were no privacy breaches or technical problems happened.

19-ii) Include qualitative feedback from participants or observations from staff/researchers

Include qualitative feedback from participants or observations from staff/researchers, if available, on strengths and shortcomings of the application, especially if they point to unintended/unexpected effects or uses. This includes (if available) reasons for why people did or did not use the application as intended by the developers.

	1	2	3	4	5	
subitem not at all important	○	○	○	○	○	essential

Does your paper address subitem 19-ii?

Copy and paste relevant sections from the manuscript (include quotes in quotation marks "like this" to indicate direct quotes from your manuscript), or elaborate on this item by providing additional information not in the ms, or briefly explain why the item is not applicable/relevant for your study

Yes, like this “More than half of intervention group mothers rated Jaundice Mobile Monitoring App as relatively or very convenient (58.1%) and relatively or very credible (51.0%) in measuring their child's jaundice. 87.1% of mothers were willing to recommend this App to people in need, and 87.7% held a positive attitude towards the necessity of promoting this App on a wide scale. In terms of satisfaction, only 11.1% of mothers showed dissatisfaction with the App. (Multimedia Appendix 4).”

DISCUSSION

22) Interpretation consistent with results, balancing benefits and harms, and considering other relevant evidence

NPT: In addition, take into account the choice of the comparator, lack of or partial blinding, and unequal expertise of care providers or centers in each group

22-i) Restate study questions and summarize the answers suggested by the data, starting with primary outcomes and process outcomes (use)

Restate study questions and summarize the answers suggested by the data, starting with primary outcomes and process outcomes (use).

	1	2	3	4	5	
subitem not at all important	○	○	○	○	○	essential

Does your paper address subitem 22-i? *

Copy and paste relevant sections from the manuscript (include quotes in quotation marks "like this" to indicate direct quotes from your manuscript), or elaborate on this item by providing additional information not in the ms, or briefly explain why the item is not applicable/relevant for your study

Yes, like this "In this smartphone-based RCT, we found that the intervention had a significant impact on the neonatal readmission for jaundice within 30 days of the initial discharge, mainly manifested as the readmission rate in intervention group was 10.5% (-15.9% to -5.0%) lower than control group. In contrast with control group, maternal anxiety symptoms induced by neonatal jaundice were also less severe in those who installed and used the Jaundice Mobile Monitoring App."

22-ii) Highlight unanswered new questions, suggest future research

Highlight unanswered new questions, suggest future research.

	1	2	3	4	5	
subitem not at all important	○	○	○	○	○	essential

Does your paper address subitem 22-ii?

Copy and paste relevant sections from the manuscript (include quotes in quotation marks "like this" to indicate direct quotes from your manuscript), or elaborate on this item by providing additional information not in the ms, or briefly explain why the item is not applicable/relevant for your study

Yes, like this "Although it can be regarded as a good auxiliary screening tool for neonatal jaundice in some cases, there is a scope to improve the acceptability of Jaundice Mobile Monitoring App. For example, the proportion of mothers who explicitly expressed satisfaction was less than 50%. The potential reason may be that the App was developed by a non-healthcare provider, leading to some concerns about the accuracy and credibility of its information. In this regard, we suggest that on the premise of ensuring the quality of jaundice screening technology, telemedicine guidance from healthcare professionals should be facilitated, and related units should optimize their publicity and promotion strategies."

20) Trial limitations, addressing sources of potential bias, imprecision, and, if relevant, multiplicity of analyses

20-i) Typical limitations in ehealth trials

Typical limitations in ehealth trials: Participants in ehealth trials are rarely blinded. Ehealth trials often look at a multiplicity of outcomes, increasing risk for a Type I error. Discuss biases due to non-use of the intervention/usability issues, biases through informed consent procedures, unexpected events.

	1	2	3	4	5	
subitem not at all important	○	○	○	○	○	essential

Does your paper address subitem 20-i? *

Copy and paste relevant sections from the manuscript (include quotes in quotation marks "like this" to indicate direct quotes from your manuscript), or elaborate on this item by providing additional information not in the ms, or briefly explain why the item is not applicable/relevant for your study

Yes, like this “Although research results have important clinical implications, several limitations should be recognized. Firstly, as we did not restrict the hospitals to which participants were readmitted, it was not possible to obtain and check all neonates' visit records from three study hospitals, so self-reported data was uniformly used in all outcomes. While some recall bias was inevitable, the short follow-up period (only 30 days) and the fact that parents usually attach importance to neonatal health and remember relatively well whether or not their child visited a doctor again, so the recall bias was minimized. Secondly, due to the unavailability of return visit records, we could not assess whether or not our intervention increased the readmission rate related to worsening neonatal hyperbilirubinemia, despite a significant drop in the overall rate. However, our intervention approaches are unlikely to delay admission to hospital for serious jaundice under the guidance of paediatricians. Finally, the trial was limited by the study site, in comparison with other reports [27,28,31], the 30-day neonatal readmission rate due to jaundice was higher in our study. This is partly due to inconsistent post-discharge follow-up period across studies, but may also be due to the high prevalence of neonatal hyperbilirubinemia in Hainan Province, China, a region with severe erythrocyte glucose 6 phosphate dehydrogenase (G6PD) deficiency [41,42]. It may affect the applicability of our findings to other populations. Therefore, it is necessary to validate the effectiveness of the intervention in a larger sample or in a cross-cultural context.”

21) Generalisability (external validity, applicability) of the trial findings

NPT: External validity of the trial findings according to the intervention, comparators, patients, and care providers or centers involved in the trial

21-i) Generalizability to other populations

Generalizability to other populations: In particular, discuss generalizability to a general Internet population, outside of a RCT setting, and general patient population, including applicability of the study results for other organizations

	1	2	3	4	5	
subitem not at all important	○	○	○	○	○	essential

Does your paper address subitem 21-i?

Copy and paste relevant sections from the manuscript (include quotes in quotation marks "like this" to indicate direct quotes from your manuscript), or elaborate on this item by providing additional information not in the ms, or briefly explain why the item is not applicable/relevant for your study

Yes, like this "The application of smartphone App in the monitoring of neonatal jaundice fit in with the widespread use of mobile technology currently, and the global Internet usage rate has reached 65.6% as of March 31, 2021[39]. The previous cost-benefit analysis found that for every 1 RMB invested in the smartphone-based, out-of-hospital screening mechanism for neonatal jaundice, 18.76 RMB was saved in the treatment cost of later neonatal jaundice[40]. The characteristics of low cost and high accessibility make Jaundice Mobile Monitoring App more practical for areas with backward medical conditions or inconvenient treatment. Meanwhile, this family-centered jaundice monitoring may contribute to alleviating the shortage of paediatric specialists, promoting the implementation of basic public health service program."

21-ii) Discuss if there were elements in the RCT that would be different in a routine application setting

Discuss if there were elements in the RCT that would be different in a routine application setting (e.g., prompts/reminders, more human involvement, training sessions or other co-interventions) and what impact the omission of these elements could have on use, adoption, or outcomes if the intervention is applied outside of a RCT setting.

	1	2	3	4	5	
subitem not at all important	○	○	○	○	○	essential

Does your paper address subitem 21-ii?

Copy and paste relevant sections from the manuscript (include quotes in quotation marks "like this" to indicate direct quotes from your manuscript), or elaborate on this item by providing additional information not in the ms, or briefly explain why the item is not applicable/relevant for your study

您的回答

OTHER INFORMATION

23) Registration number and name of trial registry

Does your paper address CONSORT subitem 23? *

Copy and paste relevant sections from the manuscript (include quotes in quotation marks "like this" to indicate direct quotes from your manuscript), or elaborate on this item by providing additional information not in the ms, or briefly explain why the item is not applicable/relevant for your study

Yes, like this “Trial Registration: China Clinical Trial Registration Center, ChiCTR2100049567; http://www.chictr.org.cn/showproj.aspx?proj=64245”

24) Where the full trial protocol can be accessed, if available

Does your paper address CONSORT subitem 24? *

Cite a Multimedia Appendix, other reference, or copy and paste relevant sections from the manuscript (include quotes in quotation marks "like this" to indicate direct quotes from your manuscript), or elaborate on this item by providing additional information not in the ms, or briefly explain why the item is not applicable/relevant for your study

Yes, like this “http://www.chictr.org.cn/showproj.aspx?proj=64245”

25) Sources of funding and other support (such as supply of drugs), role of funders

Does your paper address CONSORT subitem 25? *

Copy and paste relevant sections from the manuscript (include quotes in quotation marks "like this" to indicate direct quotes from your manuscript), or elaborate on this item by providing additional information not in the ms, or briefly explain why the item is not applicable/relevant for your study

Yes, like this “This work was supported by National Natural Science Foundation of China [grant numbers 71663016], Hainan Province Clinical Medical Center and The Excellent Talent Team of Hainan Province [No.QRCBT202121]. The funder had no role in the design and conduct of the study; collection, management, analysis, and interpretation of the data; preparation, review, or approval of the manuscript; and decision to submit the manuscript for publication.”

X27) Conflicts of Interest (not a CONSORT item)

X27-i) State the relation of the study team towards the system being evaluated

In addition to the usual declaration of interests (financial or otherwise), also state the relation of the study team towards the system being evaluated, i.e., state if the authors/evaluators are distinct from or identical with the developers/sponsors of the intervention.

	1	2	3	4	5	
subitem not at all important	○	○	○	○	○	essential

Does your paper address subitem X27-i?

Copy and paste relevant sections from the manuscript (include quotes in quotation marks "like this" to indicate direct quotes from your manuscript), or elaborate on this item by providing additional information not in the ms, or briefly explain why the item is not applicable/relevant for your study

Yes, like this "Conflicts of Interest: None declared."

About the CONSORT EHEALTH checklist

As a result of using this checklist, did you make changes in your manuscript? *

- ○ yes, major changes
- ● yes, minor changes
- ○ no

What were the most important changes you made as a result of using this checklist?

您的回答

How much time did you spend on going through the checklist INCLUDING making changes in your manuscript *

We spend 3 hours on going through the checklist INCLUDING making changes in your manuscript.

As a result of using this checklist, do you think your manuscript has improved? *

- ● yes
- ○ no
- ○ 其他:

Would you like to become involved in the CONSORT EHEALTH group?

This would involve for example becoming involved in participating in a workshop and writing an "Explanation and Elaboration" document

- ◯ yes
- ◉ no
- ◯ 其他:

清除所选内容

Any other comments or questions on CONSORT EHEALTH

您的回答

STOP - Save this form as PDF before you click submit

To generate a record that you filled in this form, we recommend to generate a PDF of this page (on a Mac, simply select "print" and then select "print as PDF") before you submit it.

When you submit your (revised) paper to JMIR, please upload the PDF as supplementary file.

Don't worry if some text in the textboxes is cut off, as we still have the complete information in our database. Thank you!

Final step: Click submit !

Click submit so we have your answers in our database!

提交 清除表单内容

切勿通过 Google 表单提交密码。

此内容不是由 Google 所创建，Google 不对其作任何担保。 举报滥用行为 - 服务条款 - 隐私权政策

Google 表单
