# Supplementary material for: Effects of a Smartphone-Based Out-of-Hospital Screening App for Neonatal Hyperbilirubinemia on Neonatal Readmission Rates and Maternal Anxiety: Randomized Controlled Trial
Source: J Med Internet Res. 2022 Nov 23;24(11):e37843. doi: 10.2196/37843 (PMC9730202; doi:10.2196/37843)
Supplement: Multimedia Appendix 2 [file jmir_v24i11e37843_app2.pdf]

Table S1. Items of the self-designed maternal anxiety for neonatal jaundice scale

Items
I lose sleep at night or have nightmares because I am worried about my child's jaundice.
I feel easily irritated until my child's jaundice subsides.
I feel nervous until my child's jaundice subsides.
I feel restless until my child's jaundice subsides.
I have no appetite until my child's jaundice subsides.
I can't concentrate on things because I am worried about my jaundiced child.
I am afraid that jaundice will threaten my child's health.
I go online for information about neonatal jaundice or keep consulting doctors and friends.
I observe the child's every move and repeatedly confirm whether the behavior was related to jaundice.
I keep an eye on the child's jaundice level and double check that it was within the normal range.
