# Supplementary material for: Effects of a Smartphone-Based Out-of-Hospital Screening App for Neonatal Hyperbilirubinemia on Neonatal Readmission Rates and Maternal Anxiety: Randomized Controlled Trial
Source: J Med Internet Res. 2022 Nov 23;24(11):e37843. doi: 10.2196/37843 (PMC9730202; doi:10.2196/37843)
Supplement: Multimedia Appendix 3 [file jmir_v24i11e37843_app3.pdf]

Table S2. The difference of demographic characteristics between participants completed and uncompleted the trial.[a]

Variables	Completed (n=1187)	Variables (n=237)	*P* values
Maternal age, median (IQR), y	29(26-32)	29(27-32)	0.69
Education level, n (%)			0.12
Junior high school or below	318 (26.8)	58 (24.5)	
High school or technical secondary school	286 (24.1)	46 (19.4)	
Junior college	281 (23.7)	57 (24.1)	
Undergraduate or above	302 (25.4)	76 (32.1)	
Residence, n (%)			0.50
Urban	808(68.1)	156(65.8)	
Rural	379 (31.9)	81 (34.2)	
Neonatal gender b, n (%)			0.37
Male	587 (41.3)	125(52.7)	
Female	598 (50.5)	112 (47.3)	
Gestational age c, median (IQR), wk	39.14(38.6-39.9)	39.14(38.6-39.9)	0.44
Birth weight d, median (IQR), g	3200(2900-3450)	3200(3000-3540)	0.07
Feeding patterns e, n (%)			0.43
Breastfeeding	893(75.3)	169 (71.3)	
Non-breastfeeding	23 (1.9)	5 (2.1)	
Mixed feeding	270 (22.8)	63 (26.6)	
Delivery method, n (%)			0.83
Natural delivery	797 (67.1)	161(67.9)	
Cesarean section	390(32.9)	76(32.1)	

[a] *P* values were obtained using 2-sided Student t-test or Wilcoxon rank sum test for continuous and $\chi 2$ test for categorical variables, all $P > .05$.

[b] Neonatal gender was unknown for 2 neonates.

[c] Gestational age was unknown for 3 neonates.

[d] Birth weight was unknown for 1 neonate.

[e] Feeding patterns was unknown for 1 neonate.
