# Supplementary material for: Effects of a Smartphone-Based Out-of-Hospital Screening App for Neonatal Hyperbilirubinemia on Neonatal Readmission Rates and Maternal Anxiety: Randomized Controlled Trial
Source: J Med Internet Res. 2022 Nov 23;24(11):e37843. doi: 10.2196/37843 (PMC9730202; doi:10.2196/37843)
Supplement: Multimedia Appendix 4 [file jmir_v24i11e37843_app4.pdf]

Table S3. Sensitivity analyses of primary and secondary outcomes

Outcomes	Unadjusted		Adjusted	
	OR (95%CI) or β (95% CI)	Difference [a] (95%CI)	OR (95%CI) or β (95% CI)	Difference [a] (95%CI)
Primary outcome: Neonates readmitted for jaundice after hospital discharge, n (%)	0.5*** (0.3 to 0.6)	10.7% (15.0% to 6.5%)	0.4*** (0.3 to 0.6)	9.2% (14.0% to 4.3%)
Secondary outcome: Maternal anxiety score due to jaundice [b], mean (SD)	-2.9*** (-3.8 to -2.1)	-3.0 (-3.8 to -2.2)	-3.0*** (-3.8 to -2.1)	-3.0 (-3.7 to -2.2)

[a] difference represents risk difference (RD) for primary outcome, mean difference (MD) for secondary outcome. Risk difference represents the absolute value of the difference in neonatal readmission rates between 2 groups. Mean difference represents the difference in mean maternal anxiety scores between 2 groups.

[b] Including mothers whose children developed jaundice symptoms after hospital discharge (412 intervention group; 408 control group).

***P<.001.
