# Supplementary material for: Effects of a Smartphone-Based Out-of-Hospital Screening App for Neonatal Hyperbilirubinemia on Neonatal Readmission Rates and Maternal Anxiety: Randomized Controlled Trial
Source: J Med Internet Res. 2022 Nov 23;24(11):e37843. doi: 10.2196/37843 (PMC9730202; doi:10.2196/37843)
Supplement: Multimedia Appendix 5 [file jmir_v24i11e37843_app5.pdf]

Table S4. Maternal Comments and attitudes towards Jaundice Mobile Monitoring APP of intervention group.

Variables	N (%)
Convenience [a]	
Very inconvenient	17(2.9)
Not very convenient	44(7.6)
Fairly convenient	188(32.4)
Relatively convenient	198(34.1)
Very convenient	133(22.9)
Credibility [b]	
Not credible	14(2.4)
Not very credible	50(8.6)
Fairly credible	220(38.0)
Relatively credible	179(30.9)
Very credible	116(20.0)
Willingness to recommend to those in need [b]	
Not recommended	14(2.4)
Not very recommended	61(10.5)
Fairly recommended	214(37.0)
Relatively recommended	168(29.0)
Highly recommended	122(21.1)
Necessity for widespread promotion [b]	
Very unnecessary	15(2.6)
Not very necessary	56(9.7)
Fairly necessary	299(51.6)
Relatively necessary	76(13.1)
Very necessary	133(23.0)
Satisfaction [b]	
Very dissatisfied	19(3.3)
Not very satisfied	45(7.8)
Fairly satisfied	292(50. 4)
Relatively satisfied	98(16.9)
Very satisfied	125(21.6)

[a] 25 mothers were missing data.

[b] 26 mothers were missing data.
